# A strategy targeting ferroptosis for mitochondrial reprogramming and intervertebral disc degeneration therapy

**DOI:** 10.7150/thno.117725

**Published:** 2025-08-16

**Authors:** Tianyi Wu, Yun Teng, Dawei Song, Yuqi Yang, Huaishuang Shen, Xiao Sun, Rui Chen, Leyu Zhao, Xianggu Zhong, Qi Yan, Junjie Niu, Jun Ge, Liang Cheng, Jun Zou

**Affiliations:** 1 Department of Orthopedic Surgery, The First Affiliated Hospital of Soochow University, Suzhou, Jiangsu 215000, China.; 2 Institute of Functional Nano & Soft Materials (FUNSOM), Jiangsu Key Laboratory for Carbon-Based Functional Materials & Devices, Soochow University, Suzhou, Jiangsu, China.

**Keywords:** ferroptosis, ceria nanoparticles, intervertebral disc degeneration, deferoxamine mesylate, ROS scavenging

## Abstract

**Background:** Intervertebral disc degeneration (IVDD) is a leading cause of low back pain, yet current therapies fail to reverse the degenerative process or restore disc function. Ferroptosis, a form of iron-dependent cell death characterized by lipid peroxidation, has been implicated in IVDD progression.

**Methods:** We synthesized Deferoxamine mesylate (DFOM)-loaded cerium oxide nanoparticles (DFOM@CeO_2_) as a novel ferroptosis-targeting therapeutic.

**Results:** DFOM@CeO_2_ exhibited dual functionality by scavenging reactive oxygen species (ROS) and chelating excess iron, thereby protecting nucleus pulposus (NP) cells from ferroptosis and extracellular matrix (ECM) degradation. DFOM@CeO_2_ demonstrated strong antioxidant capacity, effectively reducing iron accumulation and lipid peroxidation, and restoring glutathione peroxidase 4 (GPX4) expression in NP cells. Furthermore, DFOM@CeO_2_ improved mitochondrial respiratory chain function, reduce mitochondrial ROS production and prevent mitochondrial dysfunction. In a rat model of IVDD, DFOM@CeO_2_ significantly preserved disc height, reduced ECM degradation, and demonstrated superior therapeutic efficacy compared with DFOM or CeO_2_ alone. Transcriptome analysis revealed that DFOM@CeO_2_ modulates key ferroptosis-related genes and promotes mitochondrial reprogramming.

**Conclusions:** These findings highlight DFOM@CeO_2_ as a promising therapeutic strategy for IVDD, targeting both ferroptosis and mitochondrial dysfunction.

## Introduction

With the evolution of modern lifestyles, low back pain (LBP) has emerged as a prevalent clinical condition globally, ranking as the most common health issue in 134 out of 204 countries analyzed in the 2019 Global Burden of Disease Study 1, 2. Among the numerous causes of LBP, intervertebral disc degeneration (IVDD) stands out as a major contributor. Current therapeutic interventions, including conservative treatments with anti-inflammatory and analgesic medications, as well as surgical approaches, have proven inadequate in reversing the pathological process of IVDD or restoring disc tissue function 3. Consequently, basic research into LBP has increasingly focused on exploring novel strategies to ameliorate IVDD 4. The intervertebral disc consists of two main components: the nucleus pulposus (NP) and the annulus fibrosus (AF). Degeneration of the NP is widely recognized as the primary driver of IVDD, and is characterized by a decline in the extracellular matrix (ECM), which is composed of collagen II and aggrecan 5. This degradation of the ECM is often accompanied by the upregulation of metalloproteinases (MMPs), such as MMP3 and MMP13, which further facilitate ECM breakdown. However, the complete pathogenesis of IVDD remains poorly understood.

Ferroptosis, a newly recognized form of cell death, is characterized by iron-dependent lethal lipid peroxidation and has gained recognition as a widespread and evolutionarily conserved cell death mechanism 6. Ferroptosis is triggered by the presence of iron ions and is regulated by proteins, such as divalent metal ion transporter 1 (DMT1) and ferroportin 7. When iron metabolism is disrupted, leading to an accumulation of free intracellular iron, it catalyzes the Fenton reaction, generating a substantial quantity of reactive oxygen species (ROS) 8. These ROS, in turn, exacerbate lipid peroxidation and ultimately induce ferroptosis. The defense mechanisms against ferroptosis rely on cellular antioxidant systems that directly neutralize lipid peroxides. These include the GPX4-GSH system in both the cytoplasm and mitochondria, the FSP1-CoQH2 system in the cytoplasm, the DHODH-CoQH2 system in the mitochondria, and the GCH1-BH4 system, among others 9. A key aspect of ferroptosis regulation involves glutathione (GSH), a critical antioxidant. When cellular cystine transporters are inhibited, intracellular GSH becomes depleted, leading to the inactivation of glutathione peroxidase 4 (GPX4). This inactivation results in the accumulation of lipid peroxides, which, once they reach a critical threshold, can trigger cell death. Similarly, direct inhibition of the GPX4 enzyme (e.g., by RSL3) can cause this buildup of lipid peroxides, ultimately inducing ferroptosis 9. Based on our study and previous reports, ferroptosis seems to be a promising therapeutic target for the treatment of IVDD 10. However, addressing only one of the key drivers of ferroptosis, such as iron overload or ROS-mediated lipid peroxidation, is insufficient to fully mitigate the threat of ferroptosis to NP cells 11. This highlights the urgent need for an IVDD therapeutic platform that specifically targets both hallmarks of ferroptosis: iron overload and lipid peroxidation.

Currently, certain materials targeting ferroptosis have been explored for the treatment of IVDD. However, these are predominantly organic materials, which may have limitations in terms of stability when compared with inorganic nanomaterials. Additionally, the relatively complex synthesis processes of these materials' present challenges for widespread application 12, 13. Consequently, the development of an inorganic material with enhanced stability and a more straightforward synthesis approach holds promise for broader clinical and research utilization. Based on the pathological mechanism, various functional inorganic nanoparticles with ROS scavenging capabilities have been designed as novel and effective candidates for IVDD. Among these, cerium oxide nanoparticles (CeO_2_ NPs), which are characterized by the formation of oxygen vacancies and low valence states, have been extensively studied for their ability to address ROS accumulation in various diseases because of their potent antioxidant capacity 14, 15. CeO_2_ NPs are widely used owing to their low biotoxicity, exceptional biocompatibility, and enzymatic activity 16.

**Scheme 1:**
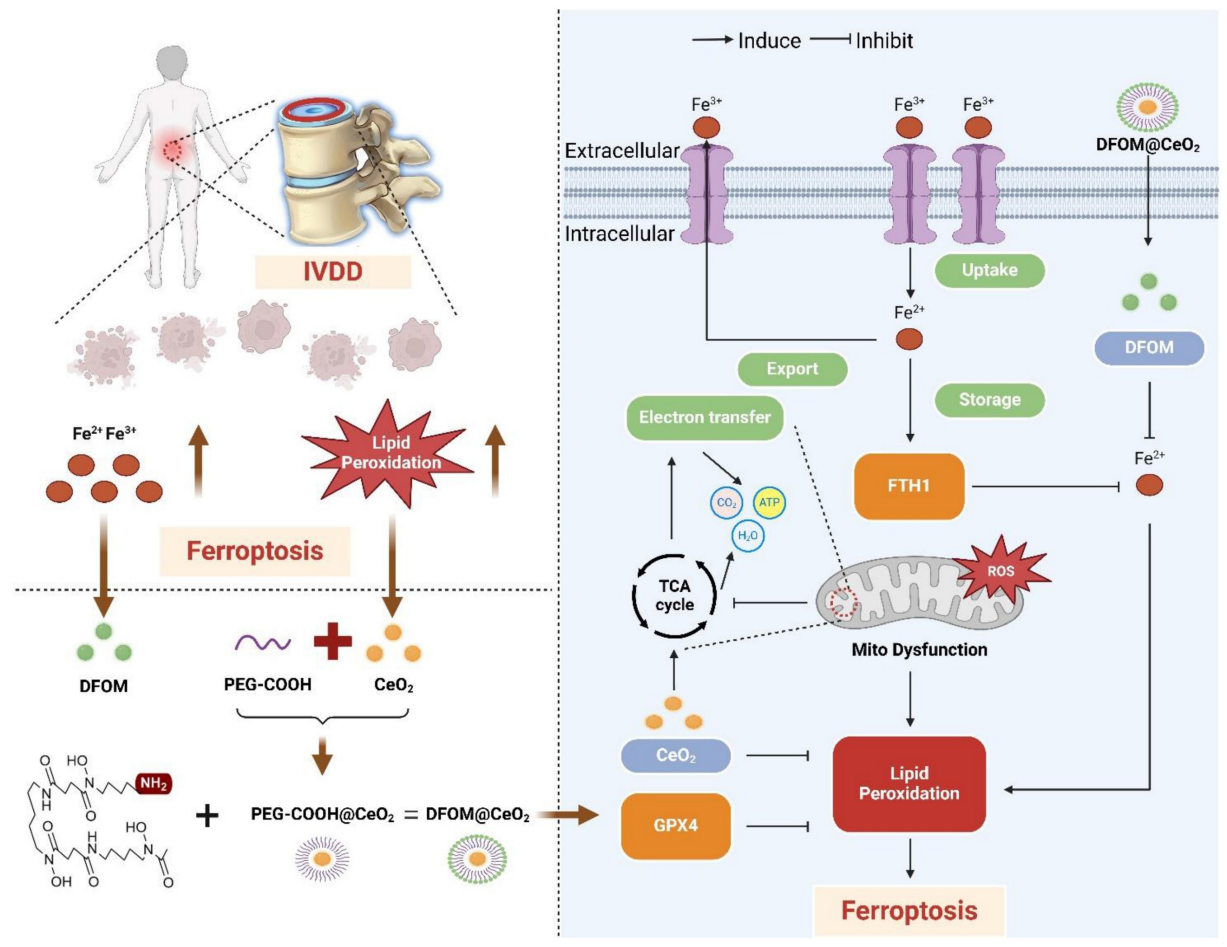
Schematic diagram of the mechanism of DFOM@CeO_2_ nanoparticles to maintain microenvironmental iron homeostasis and inhibit lipid peroxidation as a therapeutic strategy for IVDD.

Deferoxamine mesylate (DFOM), a chelating agent that binds Fe(III) and many other metal cations, is commonly used to decrease iron accumulation and deposition in tissues. As an iron chelation therapy, DFOM has entered clinical trials for treating diverse conditions, such as advanced liver cancer and cerebral hemorrhage 17, 18. CeO_2_ and DFOM exhibit promising potential in eliminating ROS-related lipid peroxidation and reducing the release of iron ions within the IVDD microenvironment, respectively, and have demonstrated the possibility of inhibiting ferroptosis. Herein, we designed a novel nanoparticle by loading DFOM onto CeO_2_ NPs (DFOM@CeO_2_) to achieve a more stable and reliable reduction effect in mitigating IVDD. CeO_2_ nanoparticles with a size of approximately 5 nm were synthesized and loaded with DFOM through carboxyl-PEGylation. These nanoparticles, possessing superior radical scavenging abilities, scavenged ROS and protected damaged mitochondria, thereby maintaining their morphology and function. In this process, it is surprising that these nanoparticles can effectively alleviate the disruption of mitochondrial homeostasis by delaying the destruction of mitochondrial respiratory chain during IVDD process. Simultaneously, they removed excess iron ions from the cellular environment, inhibiting ferroptosis (Scheme 1). Overall, our synthesis offers a novel approach for the treatment of IVDD with promising application prospects.

## Results and Discussion

### Accumulation of iron ion concentration and ferroptosis in the nucleus pulposus of patients with IVDD

**Figure 1:**
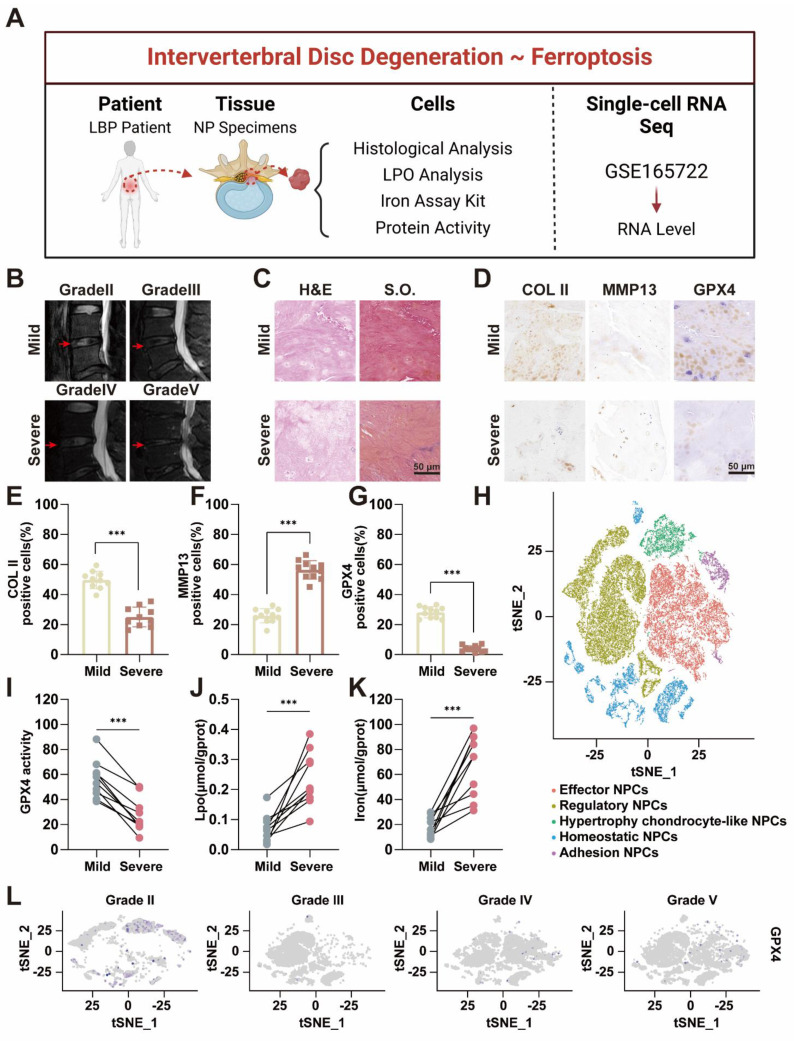
Accumulation of iron ion concentration and ferroptosis in the nucleus pulposus of patients with intervertebral disc degeneration. (A) Schematic representation of nucleus pulposus tissue obtained from a human and tested for subsequent processing. (B) MR images of patients with Pfirrmann grade II-V disc degeneration. Mild group: Grade II-III, Severe: Grade IV-V. (C) Hematoxylin-eosin (H&E) and Safranin O-fast Green (S.O.) staining of different degenerated groups. Scale bar: 50 μm. (D) Immunohistochemical analysis of COLII, MMP13, and Gpx4 staining in patient discs. Scale bar: 50 μm. (E-G) Quantitative analysis of COLII-positive cells, MMP13-positive cells, and GPX4-positive cells. (H) UMAP visualization of human nucleus pulposus cells revealed five distinct clusters through unsupervised clustering. Each dot represents an individual cell, with colors indicating the different cell clusters. (I-K) Levels of GPX4 activity, LPO, and iron ions in human nucleus pulposus tissues. (L) GPX4 immunohistochemical detection results in the nucleus pulposus tissues from different degeneration groups.

Ferroptosis requires the presence of iron ions 19, 20, and various factors influence the occurrence of ferroptosis by modulating the transport and metabolism of these ions 11, 21. Several studies have suggested the occurrence of ferroptosis in the process of IVDD 22, 23, which is mainly based on the expression of GPX4. Furthermore, previous bioinformatics analyses indicated that degenerative intervertebral discs were enriched in ferroptosis-related pathways 24. However, definitive evidence of iron accumulation and ferroptosis in IVDD remains elusive, particularly due to the lack of support from clinical samples. Our preliminary clinical sample investigation was performed to reveal the correlation between ferroptosis and IVDD (Figure 1A). Twenty NP specimens were obtained from the degenerative discs of 20 patients who underwent lumbar surgery. The patients' basic information can be found in Table S1 and Table S2 in the Supporting Information. Among these 20 patients, 8 were male and 12 were female, and there were no significant differences in baseline characteristics among the groups. The degree of NP degeneration was classified into five grades via the traditional Pfirrmann grade of magnetic resonance imaging (MRI), with Grade I defined as normal NP tissue, which was challenging to obtain because of difficulties in acquiring samples from normal patients 25. Therefore, we designated grades II-III as the mild degeneration group and grades IV-V as the severe degeneration group (Figure 1B). Based on this grouping, the 20 samples were divided into two groups, with 10 specimens in each. Histological evaluation via hematoxylin-eosin (H&E) and Safranin O-fast green (S.O.) staining demonstrated that the NP tissue in the severe group exhibited significantly more rupture, abnormal morphology, and replacement by fibrous tissue than did that in the mild group (Figure 1C). The histological grade was determined based on the extent of annulus fibrosus (AF) damage, the extent of the boundary between the AF and the NP, and the morphology of the NP cells and the NP matrix 26, 27. The results revealed that the histological grade was significantly greater in the severe group than in the mild group (Figure S1). Furthermore, the immunohistochemical results revealed that collagen II expression was much greater in the mild group than in the severe group, indicating that ECM synthesis was inhibited as the degree of degeneration increased (Figure 1D-E). Conversely, MMP13 expression was lower in the mild group than in the severe group, suggesting that ECM breakdown is enhanced as degeneration progresses (Figure 1D, F). The results of the histological evaluation of the NP tissue closely aligned with those of the radiological assessment.

Subsequently, we performed a detailed characterization of the hallmarks of ferroptosis within the NP tissue. Both the expression levels and activity of GPX4 were notably lower in the severe group than in the mild group (Figure 1D, G, I). As GPX4 plays a crucial role in reducing lipid peroxidation by converting lipid hydroperoxides to lipid alcohols, its reduced expression suggested an increased presence of reactive oxygen species (ROS) in degenerated discs 28. All these findings were further confirmed by quantitative analysis of lipid peroxidation (LPO) (Figure 1J). Compared with those in mildly degenerated NP, the contents of iron were significantly greater in severely degenerated NPs (Figure 1K). These findings reflected iron accumulation in severely degenerated NP. These results preliminarily demonstrated that ferroptosis was associated with the progression of IVDD. Based on our previous single-cell RNA sequencing (scRNA-seq) performed with the BD Rhapsody system 29, we conducted a comprehensive analysis of ferroptosis-related gene expression in all types of NPCs. GPX4 expression was significantly lower in Grade IV and V patients than in Grade II and III patients (Figure 1H, L). Ferritin, the primary intracellular iron-storage protein, consists of two subunits, FTL (ferritin light chain) and FTH1 (ferritin heavy chain 1), and there is a positive correlation between ferritin levels and tissue iron content 30. Therefore, FTH1 expression was significantly upregulated in Grade IV and V patients (Figure S2).

From human specimens to high-throughput single-cell sequencing, our results revealed significant changes in the expression of ferroptosis-related genes as IVDD progresses, which was positively correlated with the iron content in the tissue. Our study highlighted the potential role of ferroptosis in IVDD and suggested that targeting ferroptosis-related pathways could offer novel therapeutic strategies to slow or even reverse the progression of IVDD.

### Synthesis and characterization of DFOM@CeO_2_ nanoparticles

**Figure 2:**
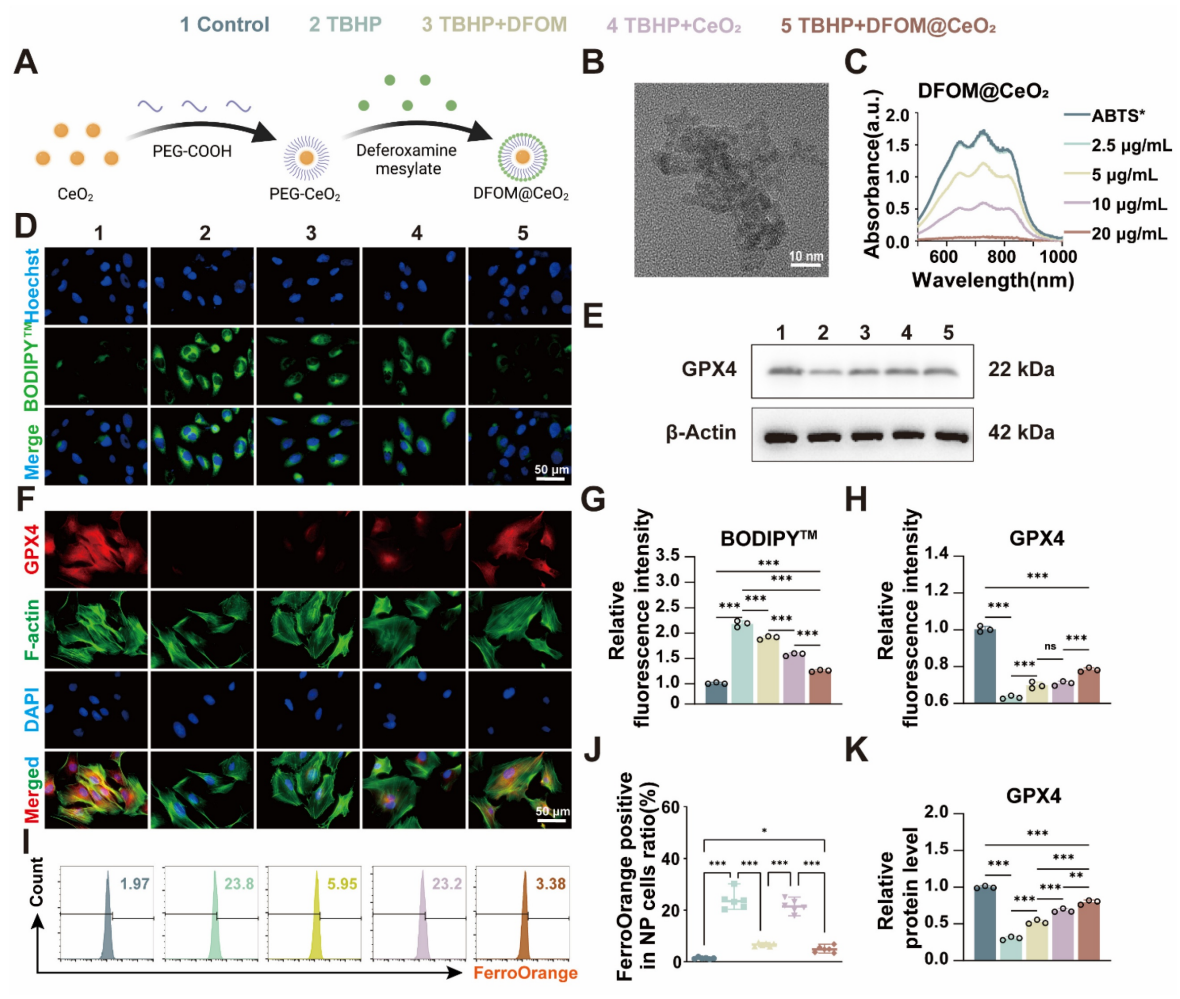
Synthesis and characterization evaluation of DFOM@CeO_2_ nanoparticles and DFOM@CeO_2_ inhibits ferroptosis in NPCs *in vitro*. (A) Schematic of the preparation of DFOM@CeO_2_. (B) Transmission electron microscopy images of CeO_2_. Scale bar: 10 nm. (C) UV-vis absorbance spectra of ABTS+• after incubation with different concentrations of CeO_2_ nanoparticles (n = 3). (D) Lipid peroxidation levels were detected by immunofluorescence staining for BODIPY^TM^ in NPCs. Scale bar: 50 μm. (E) Western blot of GPX4 in NPCs. (F) Immunofluorescence staining was used to analyze the expression of GPX4 in Scale bar: 50 μm. (n = 3). (G) Quantitative analysis of BODIPY^TM^ staining immunofluorescence (n = 3). (H) Quantitative analysis of GPX4 immunofluorescence staining (n = 3). (I) Intracellular Fe^2+^ levels were measured by flow cytometry using FerroOrange. (J) Quantitative analysis of intracellular Fe^2+^ levels (n = 3). (K) Western blot analysis and quantification of GPX4 in NPCs. (n = 3).

Previous studies predominantly focused on single-strategy approaches to counteract ferroptosis, such as inhibiting ferroptotic pathways or scavenging ROS. For instance, some achieved iron metabolism homeostasis using magnetically responsive hydrogels 31, while others stabilized mitochondrial ROS regulators 32. Though a limited number of studies adopted combined strategies targeting both iron overload and oxidative stress, their reliance on complex material systems—such as poly(lactic-co-glycolic acid) (PLGA) 12—entailed cumbersome synthesis and compositional intricacy, hindering clinical translation. Consequently, there remains an urgent need to develop readily synthesizable, multifunctional nanomaterials capable of concurrently eliminating excess iron ions and exerting robust antioxidant effects to comprehensively mitigate ferroptosis. Considering that DFOM contains free amino groups, we attempted to chemically graft DFOM onto the nanoparticles via amide bonds formed with carboxyl groups. First, CeO_2_ nanoparticles (2 nm) were synthesized via a high-temperature thermal decomposition method 15, 33. We than modified the CeO_2_ particles with carboxyl-functionalized polyethylene glycol (PEG) and finally conjugated them with DFOM to form DFOM@CeO_2_ nanoparticles (Figure 2A). Transmission electron microscopy (TEM) revealed that the synthesized CeO_2_ nanoparticles were uniform in size (~ 2 nm) and had a narrow size distribution (Figure 2B). Compared with CeO_2_, the synthesized DFOM@CeO_2_ have a little agglomeration (Figure S3A). Elemental mapping and energy-dispersive spectrometry (EDS) confirmed the uniform distribution and abundance of Ce in CeO_2_ (Figure S3B). DFOM was successfully conjugated to CeO_2_ via amide bonds, as confirmed by the characteristic absorption peak at 416 nm in the Fourier transform infrared (FTIR) spectrum (Figure S4A). After peg modification, the average hydrodynamic size of the nanoparticles was 61.9 nm. After DFOM loading, the average hydrodynamic size of the nanoparticles increased from ~61.9 nm to ~114.0 nm (Figure S4B), and the zeta potential changed from 24.4 mV to 31.9 mV (Figure S4C). With an appropriate zeta potential, the nanoparticles also demonstrated excellent stability in various media, including water, 0.9% NaCl solution, phosphate-buffered saline (PBS), and Roswell Park Memorial Institute 1640 (RPMI 1640) media, without any observable aggregation (Figure S4D). Additionally, the surface Ce^3+^/Ce^4+^ ratio in DFOM@CeO_2_ nanoparticles was quantified using X-ray photoelectron spectroscopy (XPS), with deconvolution of the Ce 3d spectra (Fig. XB) revealing a Ce^3+^ fraction of ~35% (Ce^3+^/Ce^4+^ ratio ~ 0.53), indicative of substantial oxygen vacancy formation. These vacancies confer critical biological functionality by enabling dynamic redox cycling that drives reactive oxygen species (ROS) scavenging: Ce^3+^ sites rapidly reduce superoxide (O_2_•^-^) to hydrogen peroxide (H_2_O_2_), while adjacent Ce^4+^ sites catalytically decompose H_2_O_2_ to water, collectively mimicking superoxide dismutase (SOD) and catalase activities. The results revealed an optimal Ce^3+^/Ce^4+^ ratio, which contributed to the superior antioxidant performance of DFOM@CeO_2_ (Figure S4E). Overall, ceria nanoparticles serve as optimal carriers due to combined advantages: facile synthesis via mild methods enables scalable production, superior biocompatibility guarantees biosafety in physiological systems, and most notably, the intrinsic Ce³⁺/Ce⁴⁺ redox cycle confers SOD-mimetic activity, establishing robust antioxidant defense.

To further assess the antioxidant capacity of DFOM@CeO_2_, 2,2'-azinobis-(3-ethylbenzthiazoline-6-sulfonate) and ABTS probes were used 34. The ABTS+• cationic free radical, which appears turquoise, fades to colorless in the presence of antioxidants. When mixed with DFOM@CeO_2_, the color intensity progressively decreased, and the mixture eventually became colorless as the concentration of DFOM@CeO_2_ increased (Figure 2C), confirming its antioxidant activity. The results of SOD activity also showed that the synthesized DFOM@CeO_2_ had stronger SOD activity with increasing concentration (Figure S5). By comparing the iron ion chelating efficiency, DFOM@CeO_2_ and DFOM have almost the same iron ion chelating efficiency at the same concentration (Figure S3D). Our primary goal was to demonstrate the potential of DFOM@CeO_2_ to reduce NPC degeneration and extracellular matrix degradation during IVDD. Since the endolysosomal system is crucial for the cellular uptake of nanomaterials, we investigated DFOM@CeO_2_ uptake via LysoTracker, a green fluorescent probe specific to lysosomes. DFOM@CeO_2_ was tagged with red Cy3 dye for tracking, while Hoechst staining was used to label the cell nuclei in blue. Over the course of 1 to 2 h, Cy3-labeled DFOM@CeO_2_ accumulated in the cell and partially overlapped with green lysosomal fluorescence, with red fluorescence intensifying over time. These findings suggested that DFOM@CeO_2_ is internalized via the endolysosomal pathway (Figure S4F).

The results of the CCK-8 assay and live/dead staining revealed that DFOM@CeO_2_ had no significant effect on NPCs at concentrations ranging from 0 to 10 μg/mL (Figure S4G-H)**.** And the results of CCK-8 over a longer period of time also confirmed this finding (Figure S6). Furthermore, *in vivo* studies revealed no significant differences in H&E staining of the heart, liver, spleen, lungs, or kidneys of mice injected with DFOM@CeO_2_ via the tail vein compared with those of controls (Figure S7), confirming the biocompatibility of DFOM@CeO_2_. DFOM@CeO_2_ NPs were successfully synthesized, characterized, and demonstrated excellent antioxidant activity and stability. These compounds exhibited efficient cellular uptake through the endolysosomal pathway and showed no cytotoxicity *in vitro*.

### DFOM@CeO_2_ inhibits oxidative stress and ferroptosis in NPCs *in vitro*

To further evaluate its effects *in vitro*, we used the classical tert-butyl hydroperoxide (TBHP) model to induce oxidative stress and ferroptosis in NPCs 35. Following TBHP treatment, the NPCs presented clear signs of oxidative stress and ferroptosis. On the one hand, we aimed to harness the unique antioxidant properties of CeO_2_ to mitigate TBHP-induced oxidative stress and reduce lipid peroxidation. On the other hand, the introduction of the iron chelator DFOM allowed us to target iron overload-induced ferroptosis at its source. Ultimately, our goal was to slow and even reverse the degeneration of nucleus pulposus cells. Iron overload is the primary contributor to ferroptosis. Flow cytometry using FerroOrange confirmed a significant increase in iron levels in the TBHP group, while the TBHP+DFOM@CeO_2_ group showed a marked reduction in iron accumulation (Figure 2I-J). These findings suggested that DFOM@CeO_2_ effectively chelated iron ions, reducing their accumulation in NPCs. However, the iron-chelating ability of DFOM is limited and does not fully reduce iron levels to a non-ferroptotic state 36. While DFOM effectively chelates labile iron ions, its clinical utility is constrained by three inherent limitations, including non-specific binding to physiological iron pools causing systemic toxicity, rapid oxidative degradation in ROS-rich microenvironments and insufficient capacity to fully suppress iron-catalyzed lipid peroxidation cascades. The DFOM@CeO_2_ nanoarchitecture synergistically overcomes these constraints through dual-compensatory mechanisms. CeO_2_'s catalase-mimetic activity scavenges extracellular ROS, preventing oxidative inactivation of DFOM and extending its functional half-life. Concurrently, CeO_2_ quenches lipid hydroperoxides, blocking iron-independent peroxidation propagation. This dual functionality shifts the redox balance beyond the ferroptotic threshold, achieving comprehensive protection unattainable by monotherapy.

ROS-induced lipid peroxidation, another key indicator of ferroptosis, triggers ferroptosis through damage to the cell membrane 37. To assess oxidative stress levels in NPCs, we used the BODIPY™ probe to detect lipid peroxidation products. DFOM@CeO_2_ significantly inhibited TBHP-induced lipid peroxidation in NPCs (Figure 2D, G). Although both DFOM and CeO_2_ individually reduced BODIPY™ expression, significant differences remained compared with those in the control group, indicating that a single approach may not be sufficient to effectively alleviate oxidative stress in degenerated NPCs. Similarly, the results of MDA also confirmed this effect of DFOM, CeO_2_ and DFOM@CeO_2_ (Figure S8). GPX4 is the key enzyme involved in lipid peroxidation and ferroptosis inhibition. Immunofluorescence analysis demonstrated that the DFOM@CeO_2_ formulation significantly increased the fluorescence intensity of GPX4 (Figure 2F, H). Similarly, western blot (WB) analysis confirmed that GPX4 protein levels were elevated following DFOM@CeO_2_ treatment (Figure 2E, K). Moreover, the FTH1 protein levels were also elevated following DFOM@CeO_2_ treatment (Figure S9). All these findings suggested that DFOM@CeO_2_ mitigated ferroptotic damage by chelating iron ions and simultaneously inhibited lipid peroxidation, thereby preserving GPX4 function. It is worth noting that while both DFOM and CeO_2_ individually improved oxidative stress in degenerated NPCs and significantly increased GPX4 expression, there were still significant differences compared with those in the control group. This finding suggested that a single strategy might not be sufficient to prevent ferroptosis effectively in NPCs and, consequently, improve degeneration (Figure 2D-K).

### DFOM@CeO_2_ modulates homeostasis through the mitochondrial respiratory chain in NPCs

**Figure 3:**
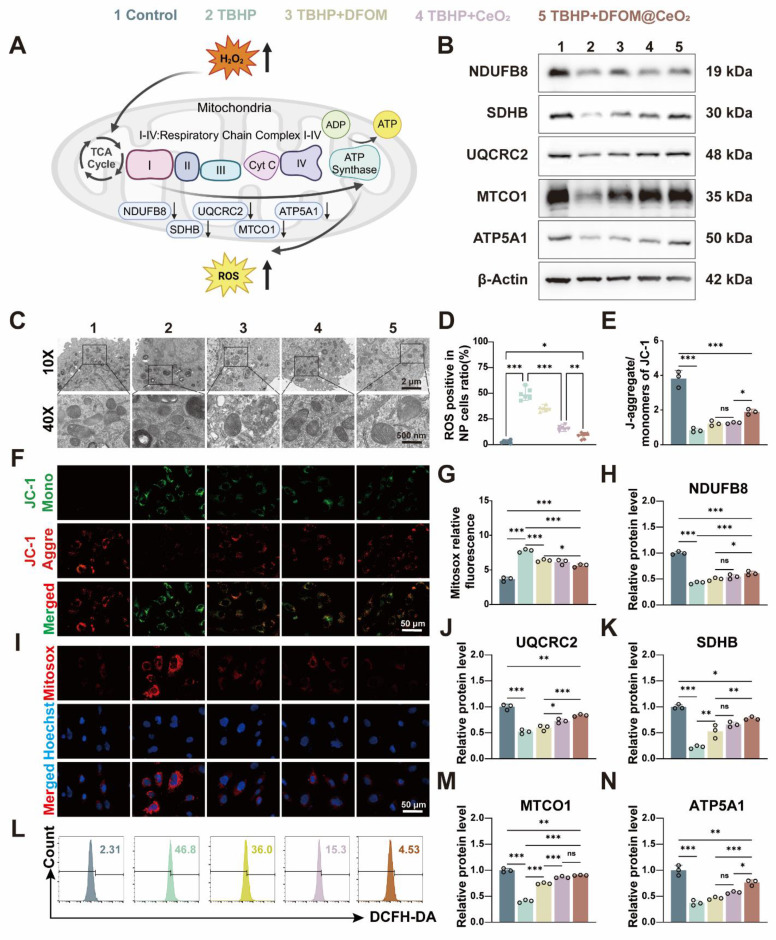
DFOM@CeO_2_ modulates ROS production through the mitochondrial respiratory chain in NPCs. (A) Schematic diagram of TBHP increasing intracellular ROS levels by inhibiting the mitochondrial respiratory chain. (B) Western blot analysis of NDUFB8, SDHB, UQCRC2, MTCO1, and ATP5A1 in NPCs. (n = 3). (C) TEM analysis of mitochondria in NPCs. Scale bar: 100 μm. (n = 3). (D) Quantitative analysis of intracellular ROS levels (n = 3). (E) Quantitative analysis of JC-1 staining (n = 3). (F) Mitochondria membrane potential of NPCs in different groups after JC-1 staining. JC-1 aggregate: green, JC-1 monomer: red. Scale bar: 50 μm. (G) Quantitative analysis of MitoSOX staining (n = 3). (H, J-K, M-N) Western blot quantification of NDUFB8, SDHB, UQCRC2, MTCO1, and ATP5A1 in NPCs. (n = 3). (I) Mitosox staining was used to analyze the amount of ROS produced by the mitochondria. Scale bar: 50 μm. (n = 3). (L) Intracellular ROS levels were measured by flow cytometry using DCFH-DA.

Though the specific mechanism remains unclear, the previous study has confirmed that DFOM@CeO_2_ can reduce lipid peroxidation levels. Mitochondria play a key role in adenosine triphosphate (ATP) synthesis and the production of ROS 38. Disruptions in mitochondrial dynamics caused by various stimuli, such as inflammation, compression, and oxidative stress, can lead to mitochondrial fragmentation and dysfunction, directly contributing to cell death 39. The mitochondrial dysfunction induced by ROS and the aging of NPC can lead to IVDD 40. Under transmission electron microscopy, NPCs in the TBHP group presented decreased mitochondrial volume, increased bilayer membrane density, reduced or absent mitochondrial cristae, ruptured and shrunken outer membranes, and dark staining of mitochondria. However, in the NPCs pre-treated with DFOM@CeO_2_ before being exposed to TBHP, the morphology of the mitochondria was restored, and the density of the mitochondrial cristae increased (Figure 3C). Next, JC-1 staining was used to measure the mitochondrial membrane potential, and Mitosox staining was used to assess the mitochondrial ROS levels. Compared with the control group, the TBHP group presented a significant decrease in the mitochondrial membrane potential and a marked increase in the ROS level, indicating mitochondrial dysfunction in TBHP-treated NPCs. This dysfunction was substantially alleviated in the DFOM@CeO_2_ group following treatment (Figure 3E-G, I). When the mitochondrial membrane potential is intact, JC-1 accumulates in the mitochondrial matrix to form polymers, emitting red fluorescence. However, when the membrane potential decreases, JC-1 cannot accumulate, resulting in green fluorescence 41. Upon entering the mitochondria, MitoSOX was oxidized by superoxide and subsequently bound to nucleic acids within the mitochondria or nuclei, producing strong red fluorescence 42. These observations suggested that TBHP treatment caused mitochondrial electron leakage, and the resulting mitochondrial dysfunction contributed to increased ROS levels. Additionally, DCFH-DA analysis revealed that DFOM, CeO_2_, and DFOM@CeO_2_ reduced the elevated ROS levels caused by TBHP to varying degrees, with DFOM@CeO_2_ resulting in the most significant reduction (Figure 3D, L).

Oxidative phosphorylation (OXPHOS) activation is associated with oxidative damage to mitochondria in ferroptotic cells 43. Thus, to further explore the mechanisms of mitochondrial dysfunction, we investigated the effects of TBHP on proteins involved in the mitochondrial respiratory chain, also known as the electron transport chain (ETC, Figure 3A). The tricarboxylic acid (TCA) cycle oxidizes acetyl-CoA into two molecules of CO_2_ within the mitochondria, producing ATP and the byproducts NADH and FADH2. Electrons from NADH and FADH2 are then transferred through the ETC to generate ATP via oxidative phosphorylation (OXPHOS), particularly through respiratory chain complexes I and II. NDUFB8, a structural component of complex I, catalyzes electron transfer from NADH to ubiquinone (CoQ), playing a crucial role in maintaining the cellular redox balance by regulating the redox states of NADH and CoQ 44. CoQ has been proven to enhance the efficiency of mesenchymal stem cell therapy for IVDD by targeting mitochondrial reactive oxygen species 45. SDHB, a component of complex II, catalyzes the conversion of succinic acid to fumarate and the reduction of FAD to FADH2, contributing to the intracellular energy balance by influencing the TCA cycle. UQCRC2, an essential subunit of complex III, is responsible for receiving electrons from CoQ and transferring them to cytochrome c, directly promoting energy release and conversion 46. MTCO1, the core subunit of cytochrome c oxidase (COX), accepts electrons from cytochrome c in the final step of the respiratory chain, transferring them to oxygen to form water, a reaction that directly influences ATP production 47. ATP5A1, a subunit of complex V, plays a crucial role in ATP synthesis, which provides energy for antioxidant molecules such as glutathione. A decrease in ATP5A1 activity reduces the ability of mitochondria to counteract ROS 48.

When mitochondrial function is impaired, increased electron leakage from mitochondrial complexes leads to increased H_2_O_2_ generation, which is catalyzed by superoxide dismutase 49. Following TBHP treatment, the protein levels of key components, including ATP5A1, SDHB, MTCO1, NDUFB8, and UQCRC2, were significantly reduced, indicating mitochondrial damage. At the mRNA level, TBHP significantly inhibited the expression of mitochondrial complexes related genes. When DFOM, CeO_2_, and DFOM@CeO_2_ were added for treatment, this inhibitory effect was alleviated, with DFOM@CeO_2_ most significantly alleviated this inhibitory effect (Figure S10). Treatment with DFOM, CeO_2_, or DFOM@CeO_2_ restored these protein levels to varying degrees, with DFOM@CeO_2_ showing the most pronounced effect (Figure 3B, H, J-K, M-N). The Oxygen consumption rate (OCR) results showed that TBHP intervention led to mitochondrial aerobic respiration inhibition, while DFOM@CeO_2_ alleviated this inhibitory effect (Figure S3C). The dihydroorotate dehydrogenase (DHODH)-CoQH2 regulatory system can inhibit mitochondrial lipid peroxidation, thereby preventing ferroptosis 50, 51. This system, located on the cell membrane, utilizes NADP to reduce ubiquinol to CoQH, which then acts as a lipophilic radical-trapping antioxidant, preventing lipid peroxidation on the cell membrane and ultimately inhibiting ferroptosis 50. It could be found that a significant increase in NDUFB8 expression was observed compared with that in the TBHP group.

Given the role of NDUFB8 in transferring electrons from NADH to CoQ, we speculated that the DHODH-CoQH2 regulatory system represented an additional mechanism through which DFOM@CeO_2_ exerts its effects. Our findings suggested that DFOM@CeO_2_ can partially inhibit the disruption of mitochondrial function, contributing to the preservation of mitochondrial integrity and reducing the negative effects of oxidative stress in NPCs. The iron chelation mediated by DFOM blocks the Fenton reaction, directly alleviating mitochondrial membrane lipid peroxidation. CeO₂ removes superoxide anions (O₂·⁻) to protect the ETC complex, restoring the efficiency of ATP synthesis. The two pathways synergistically upregulate the expression of GPX4, forming a positive feedback mechanism against ferroptosis. Our strategy aligns with other studies. For instance, the synergistic effect of the continuous release of polysulfides by the graystone nanozyme and the antioxidant enzyme activity ultimately rescues the damaged mitochondria from functional disorders 52.

### DFOM@CeO_2_ reverses extracellular matrix degeneration in NPCs

**Figure 4:**
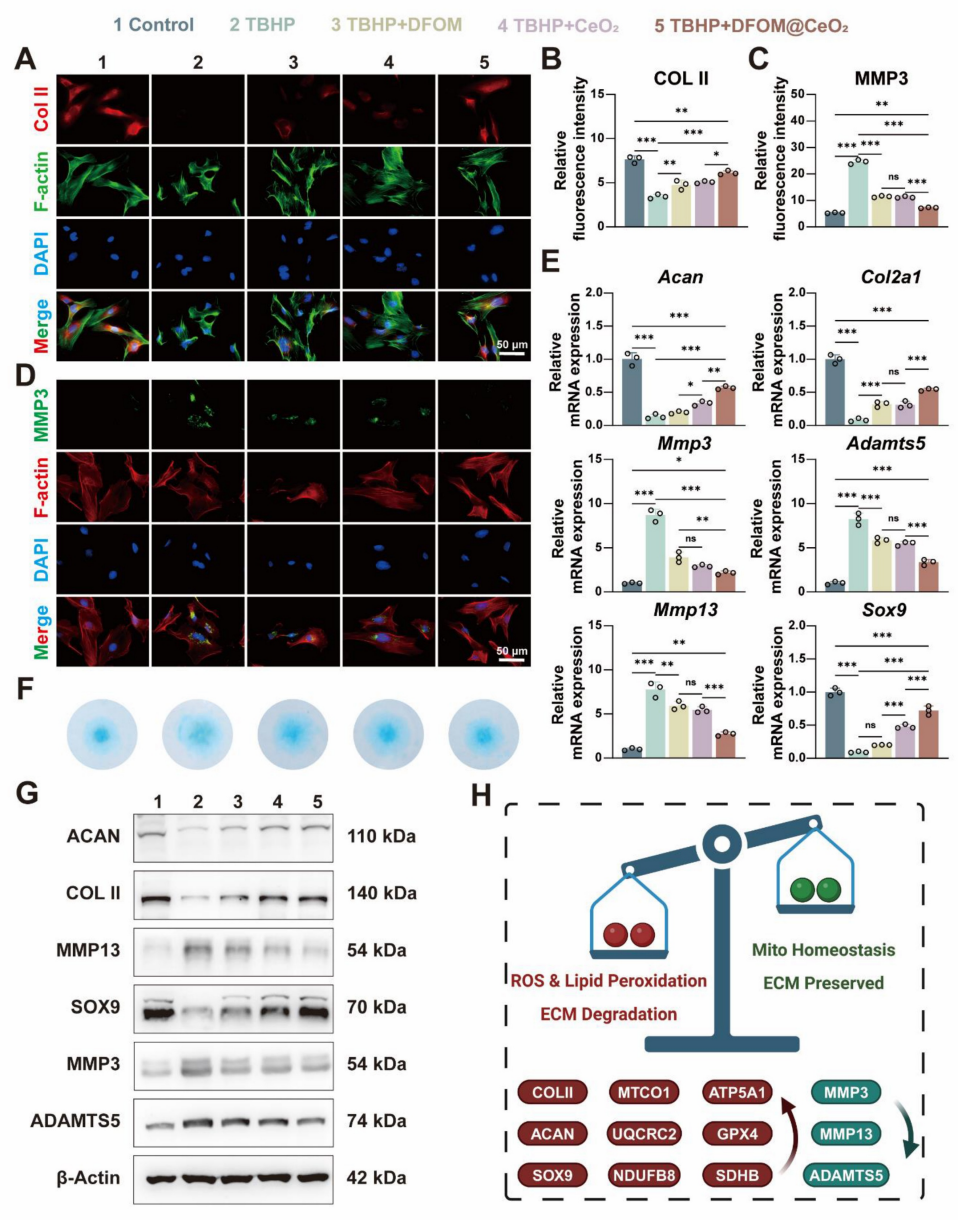
DFOM@CeO_2_ reverses extracellular matrix degeneration in NPCs. (A) Immunofluorescence staining was used to analyze the expression of COLII in NPCs treated with TBHP, TBHP+DFOM, TBHP+CeO_2_, or TBHP+ DFOM@CeO_2_. Scale bar: 50 μm (n = 3). (B) Quantitative analysis of COLII staining immunofluorescence. (C) Quantitative analysis of MMP3 staining immunofluorescence. (D) Immunofluorescence staining was used to analyze the expression of MMP3 in NPCs treated with TBHP, TBHP+DFOM, TBHP+CeO_2_, or TBHP+DFOM@CeO_2_. Scale bar: 50 μm (n = 3). (E) RT-qPCR results showing the mRNA expressions of *Acan*, *Col2a1*, *Mmp13*, *Sox9*, *Adamts5* and *Mmp3* in NPCs treated with or without TBHP, TBHP+DFOM, TBHP+CeO_2_, or TBHP+ DFOM@CeO_2_ (n = 3). (F) Alcian blue staining of NPCs cultured after treatment with TBHP, TBHP+DFOM, TBHP+CeO_2_, or TBHP+ DFOM@CeO_2_. (G) Western blot analysis of ACAN, COLII, MMP13, SOX9, ADAMTS5, and MMP3 in NPCs (n = 3). (H) Schematic diagram of the homeostasis mechanism of ROS clearance and lipid peroxidation downregulation while mitochondrial homeostasis and extracellular matrix restoration.

The above experiments confirmed the strong iron-binding and antioxidant capabilities of DFOM@CeO_2_, with its primary mechanism being the reconstruction of mitochondrial function. Next, we aimed to explore whether DFOM@CeO_2_ could mitigate ECM degradation and ultimately IVDD under ferroptotic conditions. After TBHP treatment, the mRNA expression levels of matrix degradation-related genes (*Mmp3*, *Mmp13*, and *Adamts5*) were significantly elevated, while those of matrix synthesis-related genes (*Acan*, *Col2a1,* and *Sox9*) were notably downregulated in NPCs. DFOM@CeO_2_ treatment significantly reduced the expression of ECM degradation genes and restored the mRNA levels of matrix synthesis-related genes to more than half of their normal levels (Figure 4E). These findings were further corroborated by corresponding changes in protein expression. Immunofluorescence staining revealed a reduction in red fluorescence for Collagen II and an intensification of green fluorescence for MMP3 in the TBHP group. Treatment with DFOM, CeO_2_, or DFOM@CeO_2_ reversed these effects, with DFOM@CeO_2_ exhibiting the most significant reversal but not full restoration to normal levels (Figure 4A-D). Additionally, Alcian blue staining demonstrated that DFOM@CeO_2_ promoted the secretion of extracellular matrix collagen. Compared with TBHP group, DFOM@CeO_2_ group effectively mitigated the reduction in extracellular matrix secretion (Figure 4F). Finally, WB analysis further verified the changes in protein expression levels associated with extracellular matrix degradation and synthesis (Figure 4G and Figure S11). Although DFOM@CeO_2_ did not fully return the gene and protein levels to normal levels, they substantially improved compared with those in the TBHP group. Erastin is a classic inducer of ferroptosis in cells. Surprisingly, in the Erastin-induced model, we observed that DFOM@CeO_2_ also has excellent antioxidant effects (Figure S12A, C) and effectively alleviates the destruction of extracellular matrix in NPCs. After Erastin treatment, the mRNA expression levels of matrix degradation-related genes (*Mmp3*, *Mmp13*, and *Adamts5*) were significantly elevated, while those of matrix synthesis-related genes (*Acan*, *Col2a1,* and *Sox9*) were notably downregulated in NPCs, while these effects were significantly alleviated after the intervention of DFOM@CeO_2_ (Figure S12B). The immunofluorescence results confirmed this effect at the protein level (Figure S12D-G). These were also confirmed in the WB results (Figure S12H-I). Moreover, the protein content expression of the ferroptosis key protein GPX4 in the Erastin+DFOM@CeO_2_ group was significantly higher than that in the Erastin group, which is consistent with previous findings (Figure S12H-I). These findings supported the role of DFOM@CeO_2_ in mitigating ECM degradation and potentially slowing the progression of IVDD under ferroptotic conditions.

### DFOM@CeO_2_ effectively treats intervertebral disc degeneration *in vivo*

**Figure 5:**
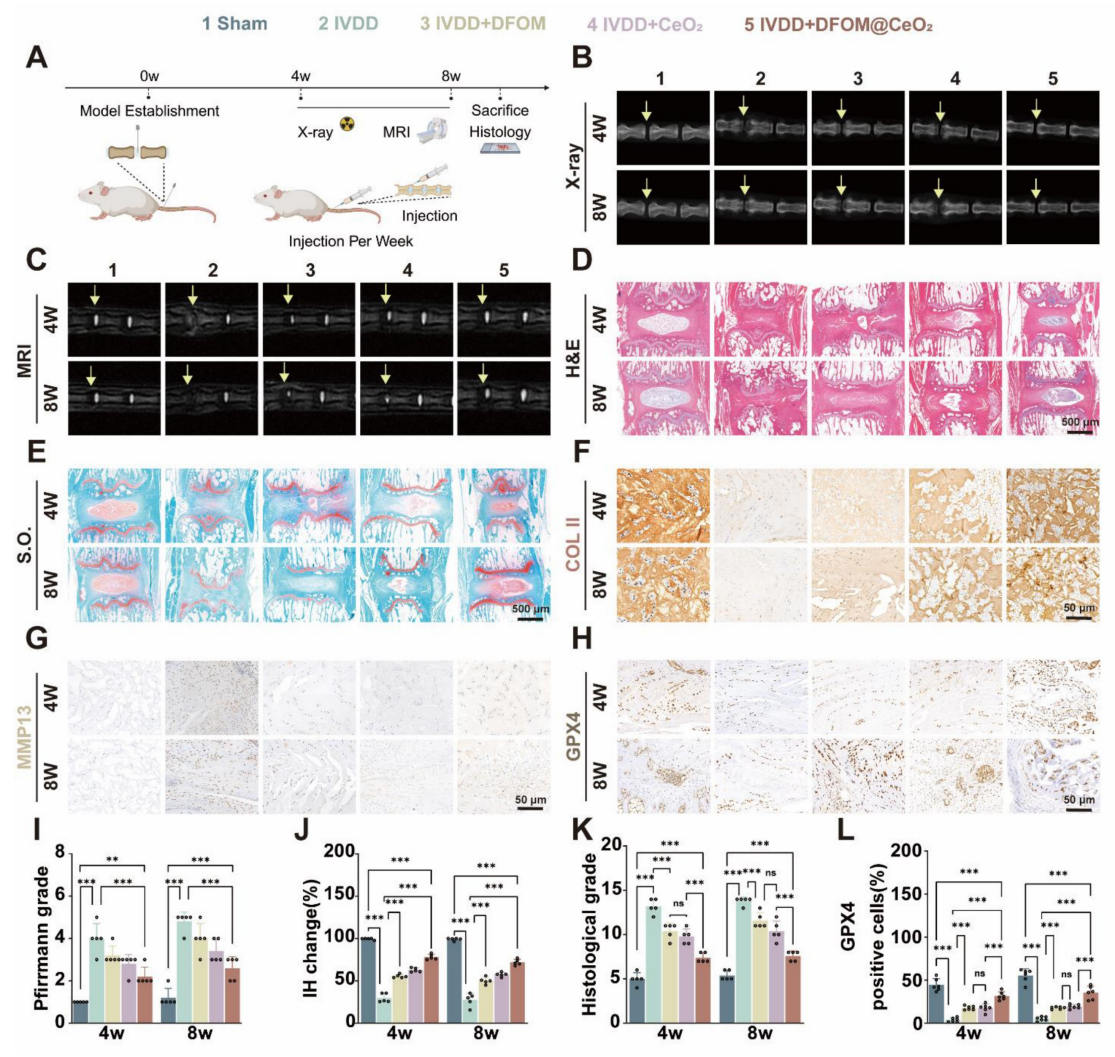
DFOM@CeO_2_ effectively treats intervertebral disc degeneration *in vivo*. (A) Schematic diagram of the animal experiment. (B) X ray images of the IVDs of the rat tail in the five groups (Sham, IVDD, IVDD+DFOM, IVDD+CeO_2_, and IVDD+DFOM@CeO_2_) at the 4^th^ and 8^th^ weeks. Yellow arrows: puncture sites. (C) MR images of the IVDs of the rat tail in the five groups (Sham, IVDD, IVDD+DFOM, IVDD+CeO_2_, and IVDD+DFOM@CeO_2_) at the 4^th^ and 8^th^ weeks. Yellow arrows: puncture sites. (D-E) Hematoxylin-eosin (H&E) and Safranin O-fast green (S.O.) staining of the five groups at the 4^th^ and 8^th^ weeks. Scale bar: 500 μm. (F-H) Immunohistochemical analysis of COLII, MMP13, and GPX4 staining of the rat tail in the five groups (Sham, IVDD, IVDD+DFOM, IVDD+CeO2, and IVDD+DFOM@CeO2) at the 4^th^ and 8^th^ weeks. Scale bar: 50 μm. Scale bar: 50 μm. (I) Pfirrmann grade of the IVDs of the rat tail in five groups at the 4^th^ and 8^th^ weeks. (J) Intervertebral heights of the IVDs of the rat tail in the five groups at the 4^th^ and 8^th^ weeks. (K) Histological grade scores of Hematoxylin-eosin (H&E) staining of the IVDs at the 4th and 8th weeks. (L) Quantitative analysis of GPX4-positive cells in the five groups at the 4^th^ and 8^th^ weeks (n = 5).

Given the excellent *in vitro* therapeutic effect of DFOM@CeO_2_, we established a rat model of IVDD to further assess its efficacy *in vivo*. The IVDD model was created via needle puncture of the caudal intervertebral disc in rats, and then the rats were divided into four groups: one group received no injections (IVDD group), while the other three groups received weekly injections of DFOM, CeO_2_, or DFOM@CeO_2_ directly into the intervertebral discs for 2 weeks. There was also a sham-operated group that did not exhibit intervertebral disc degeneration (namely, the sham group). X-rays and MRI scans were performed at 4 and 8 weeks to evaluate treatment outcomes (Figure 5A). In the IVDD sham group, X-rays revealed a decrease in intervertebral disc height, and T2-weighted MR images revealed a decrease in signal intensity, indicative of water loss in the disc. The signal intensity decreased to varying degrees in all the experimental groups over time. However, compared with the IVDD control group, all the injected groups presented varying degrees of restoration of intervertebral disc height. The MRI results revealed that the DFOM@CeO_2_ group demonstrated a stronger signal intensity than both the DFOM and CeO_2_ groups did, indicating superior *in vivo* efficacy (Figure 5B-C, I-J).

H&E staining and safranin O-fast green (S.O.) staining were used to assess the nucleus pulposus tissue after treatment for 4 and 8 weeks. S.O. staining at both time points revealed a distinct boundary between the nucleus pulposus and annulus fibrosus tissues in the control group, with safranin O staining predominant in the nucleus pulposus and cartilage endplate, while the lamellar annulus fibrosus was stained primarily with fast green. In contrast, the IVDD group exhibited an unclear boundary between the nucleus pulposus and cartilage endplate, with a significant reduction in the safranin O-stained area, which was mostly replaced by a lighter green color, along with a notable decrease in the intervertebral disc height. Remarkably, in the DFOM@CeO_2_ group, a clear boundary between the nucleus pulposus and cartilage endplate was observed, and the safranin O-stained area was significantly larger than that in the IVDD group (Figure 5D-E). Histological grading further showed that the IVDD group had the highest degeneration scores while the DFOM@CeO_2_ group presented the lowest scores consistent with the results obtained from the healthy control group (Figure 5K and Figure S13A). Furthermore, we performed immunohistochemical staining analysis at 4 and 8 weeks to assess the expression of Collagen II, MMP13, and GPX4 (Figure 5F-H). Collagen II and MMP13 are markers of nucleus pulposus matrix metabolism, while GPX4 is a key regulator of ferroptosis. These analyses further validated the *in vivo* function and mechanism of DFOM@CeO_2_. At both 4 and 8 weeks, Collagen II expression decreased across all the model groups compared with that in the control group. However, the DFOM@CeO_2_ group presented the highest level of collagen II expression, which was consistent with the above *in vitro* results (Figure S13B). In contrast, MMP13 expression increased in all the model groups, with the DFOM@CeO_2_ group showing the lowest MMP13 levels, which again aligns with the *in vitro* results (Figure S13C). Similarly, GPX4 expression decreased in all the model groups. At 4 weeks, GPX4 expression in the DFOM@CeO_2_ group returned to approximately 70% of the normal level, but this percentage slightly decreased to approximately 50% by 8 weeks (Figure 5L). DFOM@CeO_2_ demonstrated *in vivo* significant therapeutic effects by preserving extracellular matrix integrity in a rat IVDD model. Both MRI and histological evaluations confirmed that DFOM@CeO_2_ outperformed both DFOM and CeO_2_ alone in maintaining disc height and reducing degeneration markers. Additionally, DFOM@CeO_2_ partially restored GPX4 levels, indicating its potential to inhibit ferroptosis and protect against oxidative damage. These findings suggested that DFOM@CeO_2_ had great potential as a novel treatment strategy for delaying the progression of IVDD.

### Transcriptome analysis reveals the *in vivo* protective mechanisms of DFOM@CeO_2_

**Figure 6:**
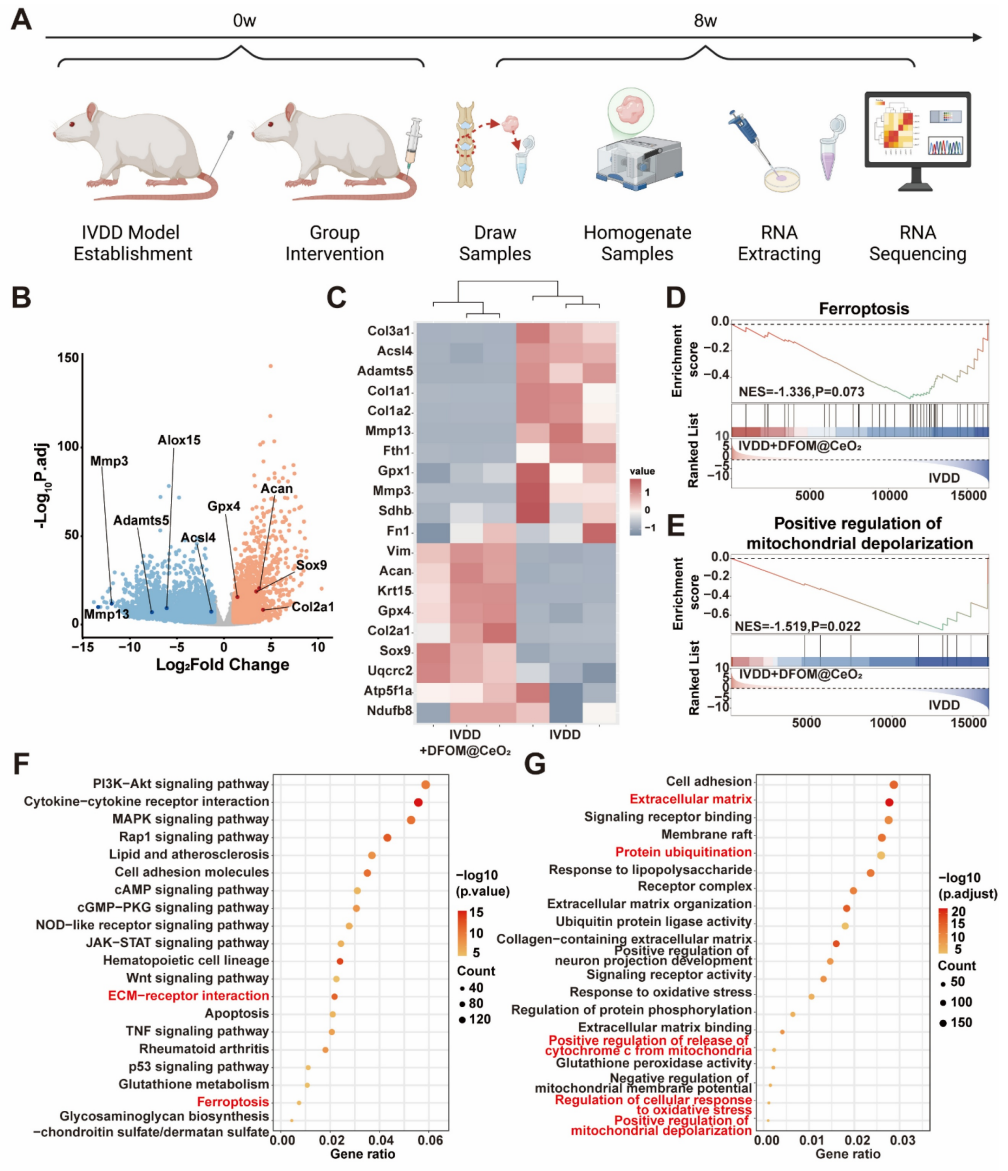
Transcriptome analysis reveals DFOM@CeO_2_'s protective mechanisms and pathway enrichment *in vivo*. (A) Schematic diagram of sample sequencing from an animal model of IVDD. (B) Volcano plots of differentially expressed genes after sequencing. (C) Heat maps of ferroptosis-related gene expression, extracellular matrix-related gene expression, and mitochondria respiratory chain-related gene expression. (D) Ferroptosis GSEA enrichment analysis based on KEGG datasets. (E) Positive regulation of mitochondrial depolarization GSEA enrichment analysis based on GO datasets. (F) KEGG enrichment analysis of signaling pathways. (G) GO enrichment analysis of signaling pathways.

To further validate the protective mechanism of DFOM@CeO_2_, we performed transcriptome sequencing analysis on NP tissue from both the IVDD group and the IVDD+DFOM@CeO_2_ group (Figure 6A). The volcano plot revealed significantly expressed genes between the two groups, and PCA confirmed robust consistency within each group (Figure S14). Genes associated with extracellular matrix degradation, such as *Mmp3*, *Mmp13*, and *Adamts5*, as well as ferroptosis-promoting genes, such as *Alox15* and *Acsl4*, were significantly downregulated in the DFOM@CeO_2_ group. In contrast, genes involved in matrix synthesis, including *Col2a1* and *Acan*, and those related to ferroptosis inhibition were upregulated (Figure 6B). The heatmap revealed that key genes involved in the mitochondrial respiratory chain, such as *UQCRC2*, *ATP5F1A*, and *NDUFB8*, were upregulated following treatment, further supporting the protective effects on the respiratory chain demonstrated in the *in vitro* experiments (Figure 6C). In addition to the consistency with the volcano plot findings, the heatmap revealed broader improvements in mitochondrial function, indicating enhanced OXPHOS capacity. These changes suggested that DFOM@CeO_2_ not only protected against ferroptosis but also contributed to the restoration of the mitochondrial metabolic balance, potentially playing a key role in maintaining cellular energy homeostasis in IVDD.

GO enrichment analysis revealed that ferroptosis (Figure 6D), the regulation of mitochondrial depolarization (Figure 6E), and ECM (Figure S15) were strongly correlated with DFOM@CeO_2_ treatment in IVDD. Additionally, through KEGG and functional enrichment analyses, we found that DFOM@CeO_2_ was closely associated with the PI3K-AKT pathway, ECM-receptor interactions, ferroptosis, and inflammatory pathways, such as the MAPK, p53, and TNF pathways (Figure 6F). The functions of these pathways were also linked to processes such as protein ubiquitination and cell adhesion, providing valuable insights for future research into the biological mechanisms of inorganic nanoparticles (Figure 6G). These results highlighted DFOM@CeO_2_ as an effective and integrated strategy for targeting ferroptosis and IVDD, which not only inhibited ECM degradation but also promoted mitochondrial reprogramming and regulated key pathways.

## Conclusion

In summary, we developed an integrated strategy targeting ferroptosis for the treatment of intervertebral disc degeneration (IVDD) by synthesizing DFOM-loaded inorganic nanoparticles (DFOM@CeO_2_). These nanoparticles exhibited strong antioxidant and iron-chelating properties, effectively inhibiting ferroptosis and extracellular matrix degradation in nucleus pulposus cells. Notably, DFOM@CeO_2_ improved mitochondrial respiratory chain function and reduced mitochondrial ROS production and lipid peroxidation. This mitochondrial reprogramming suggested that restoring respiratory chain activity might be the key to preventing IVDD progression. Despite its use as a conventional material, the innovative application of DFOM@CeO_2_ in ferroptosis inhibition and mitochondrial reprogramming demonstrated significant translational potential. This dual action of antioxidant defense and iron chelation, combined with the ability of DFOM@CeO_2_ to restore mitochondrial function, made DFOM@CeO_2_ a promising therapeutic candidate for IVDD treatment. The efficacy *in vitro* and *in vivo* showed its potential for clinical application. Further research into the long-term effects, biocompatibility, and potential synergies of DFOM@CeO_2_ with existing therapies would be essential for identifying the role of DFOM@CeO_2_ as a transformative treatment option for IVDD.

## Experimental Section

### Materials and reagents

Cerium acetylacetonate hydrate (Shanghai Yuanye Biotechnology, Shanghai, China), deferoxamine mesylate (Aladdin, Shanghai, China), oleylamine (OM), octadecene (ODE), cyclohexane, ethanol, dichloromethane, phospholipid polyethylene glycol (Ponsure biological, China), 2,2'-Azinobis-(3-ethylbenzthiazoline-6-sulphonate) (ABTS), glutathione peroxidase 4 (GPX4) activity assay kit (Elabscience, Wuhan, China), lipid peroxide (LPO) content(Solarbio, Beijing, China), assay kit iron assay kit-colorimetric (Dojindo, Japan), cell counting kit-8 (CCK-8), JC-1 staining kit, CM-H2DCFDA eyotime, Shanghai, China), MitoSOX Red kit, BODIPY 581/591 C11(MedChemExpress, Monmouth Junction, USA), FerroOrange probe (Dojindo, Japan), primary antibodies against Aggrecan, Mmp13 (Proteintech, Wuhan, China), collagenII (ABclonal, Wuhan, China), SOX9, MMP3, ADAMTS5 (abcam, Cambridge, UK), UQCRC2, SDHB, NDUFB8, MTCO1, ATP5A1, GPX4 (Huabio, Hangzhou, China), primary antibodies against β-actin (Proteintech, Wuhan, China). Goat Anti-Rabbit IgG H&L (HRP), Goat Anti-Mouse IgG H&L (HRP) (abcam, Cambridge, UK). FastPure Cell/Tissue Total RNA lsolation Kit V2, HiScript IV RT SuperMix for qPCR (+gDNA wiper), Taq Pro Universal SYBRqPCR Master Mix (Vazyme, Nanjing, China), Phosphate-buffered saline (PBS), LIVE/DEAD Cell Imaging Kit (488/570), fetal bovine serum (FBS), DMEM/F12 (Thermo Fisher Scientific, Waltham, USA).

### Tissue collection of degraded NPs in humans

We collected NPs from patients with Grade II to V degeneration according to the Pfirrmann disc degeneration scoring criteria based on the preoperative MRI findings. The basic information of the 20 patients (Table S1) revealed that 5 patients with grade II-V were enrolled each, and all patients were IVDD patients who underwent (**Posterior Lumbar Interbody Fusion**, PILF) surgery. Written informed consent was obtained from the patients or their relatives before tissue collection. This study was approved by the Ethics Committee of the First Affiliated Hospital of Soochow University (Approval No.2024550). We defined grades II to III as mild degeneration and grades IV to V as severe degeneration.

### GPX4 activity, LPO, iron detection in human NPs

Tissue samples: Homogenate was extracted according to the ratio of tissue sample mass (mg): working liquid volume (μL) =1:9 (10000 rpm, 10 min, 4 °C). The supernatant was taken for testing, and part of the supernatant was retained for protein concentration determination. Then, GPX4 activity, LPO levels, and Iron content were measured according to the manufacturer's instructions.

### Single-cell RNA-seq library construction and sequencing of human NPs

A copy of previously published scRNA-seq data (GSE165722) using the BD Rhapsody system was further analyzed via R software using the Seurat package 29. The single cell number and principal cell clusters were determined via the unsupervised FindClusters function in the Seurat package and visualized in the 2D model via the Unified Manifold Approximation and Projection (UMAP) method. The cell clusters were annotated via the FindAllMarkers function. Subsequently, chondrocyte colonies were harvested for further analysis.

### Synthesis of DFOM@CeO_2_

The process begins with 1 mmol of cerium acetonate hydrate, which is carefully dissolved in 5 mL of organic medium (OM) via ultrasonication until it is completely integrated. Moreover, a mixture of 15 mL of OM and 15 mL of octadecene (ODE) was combined and heated up to a sizzling 280 °C. Once the temperature was reached, the previously dissolved cerium acetonate hydrate was introduced slowly into this hot mixture. This reaction was allowed to simmer at 280 °C for an hour before being cooled down to room temperature. To extract the ceria nanoparticles (CeO_2_ NPs), the mixture underwent a series of washes and centrifugations using cyclohexane and ethanol. After this, the nanoparticles are dissolved in dichloromethane, where an excess of polyethylene glycol (PEG) is added under ultrasonic conditions. This mixture was sonicated for 1 h, after which the organic solvent was evaporated via rotary heating. Next, ultrapure water was added to re-dissolve the nanoparticles, and a centrifugation step was performed to eliminate any remaining PEG, resulting in the formation of PEG-coated CeO_2_ NPs. In the final step, DFOM is introduced dropwise to this aqueous solution of PEG-coated CeO_2_ NPs while ultrasonication is applied. The mixture is gently shaken overnight at room temperature, allowing the DFOM to incorporate. The end product, DFOM-loaded CeO_2_ NPs (DFOM@ CeO_2_ NPs), was isolated by centrifugation to remove any unbound DFOM molecules.

### Characterization

The morphology of the CeO_2_ NPs was characterized via transmission electron microscopy (TEM, JEOL, JEM-F200, Japan). The characteristic absorption peaks of the CeO_2_ NPs, DFOM, and DFOM@CeO_2_ NPs were measured by Fourier transform infrared spectroscopy (Thermo Fisher Scientific Nicolet iS20, USA). The zeta potential and hydrodynamic size of CeO_2_ NPs and DFOM@CeO_2_ NPs were measured via dynamic light scattering (DLS; Malvern Zetasizer Nano ZS90, USA). X-ray photoelectron spectroscopy (XPS) was performed using an ESCALAB 250Xi instrument (Thermo Scientific K-Alpha, USA).

### Free radical scavenging test

ABTS (2,2'-azinobis-(3-ethylbenzthiazoline-6-sulphonate)) was utilized to assess the free radical scavenging activity of CeO_2_ NPs. To generate ABTS+•, 0.8 mL of ABTS solution (4 mg/mL) was incubated overnight with 1 mL of potassium persulfate solution (1 mg/mL). Following this, 50 µL of the ABTS+• solution was added to 1 mL of DFOM@CeO_2_ NPs solution at various concentrations, and the absorbance of ABTS+• was measured at 734 nm.

### Biodistribution of Cy3-labeled DFOM@CeO_2_ in NPCs

To track the biodistribution of DFOM@CeO_2_, the DFOM@CeO_2_ particles were conjugated with Cy3. The uptake of DFOM@CeO_2_ in NPCs was observed at 0 h, 1 h, and 2 h.

### Cell viability assay (CCK8 and Live/dead staining)

NPCs in the logarithmic growth phase were seeded in 96-well plates at 2x10^3^ cells/well. After 24 h, the cells were completely attached to the wall, and 8 groups of complete media containing 0, 0.1, 0.5, 1, 2, 5, 10, 20 μg/mL DFOM@CeO_2_ were prepared. More than 200 μL of eight groups of complete medium containing different concentrations of DFOM@CeO_2_ were added, and each group was set up with six complex Wells. After 24 h, 48 h, and 72 h of culture, the original medium was removed, and 100 μL of α-MEM and 10 μL of CCK-8 solution were added to each well. The cell-free wells were used as the blank group, and the 0 mM DFOM group was used as the control group. The absorbance of each well was detected at a wavelength of 450 nm, and the cell proliferation rate was calculated as (OD experiment-OD blank group)/ (OD control-OD blank group) *100%. NPCs were plated in 24-well plates and co-cultured with PBS, DFOM, CeO_2,_ or DFOM@CeO_2_ at a concentration of 20 μg/mL. After washing with PBS, calcein/PI staining dye was added, and the cells were incubated at 37 °C for 30 mins at intervals of 24 h, 48 h, and 72 h. Following incubation, the cells were washed again and examined under a fluorescence microscope.

### The level of intracellular iron ions

The cells were inoculated in 6-well plates, and after cor-responding treatment with drugs, FerroOrange working mixture (1 µmol/L) was added to the 6-well plates, which were subsequently incubated for 30 min, washed with PBS three times, and then subjected to flow cytometry for iron ion level determination.

### Lipid peroxidation detection

After drug treatment in 24-well plates for 24 h, the culture medium was discarded. The cells were dissociated by adding trypsin to make a single-cell suspension. The cells were subsequently centrifuged (1000 g, 5 min, 4 °C), after which the supernatant was discarded. The samples were washed with PBS for 5 mins each. 1 mL of BODIPY 581/591 C11 working solution was added, and the mixture was incubated for 30 min at room temperature. The samples were centrifuged (400 g, 3 min, 4 °C), and the supernatant was discarded. The cells were washed twice with PBS for 5 min each. The cells were resuspended in PBS, and the fluorescence changes in each well were observed under a fluorescence microscope, and the relative fluorescence intensity was calculated via Image J software.

### Total ROS assay

The cells were inoculated in 6-well plates, and after corresponding treatment with drugs, DCFH-DA was diluted in serum-free medium at a ratio of 1:1000 to give a final concentration of 5 µM. The cell culture medium was removed, and 1 mL of diluted DCFH-DA was added. The cells were incubated in a cell incubator at 37 ºC for 30 min. The cells were washed three times with serum-free cell culture medium to adequately remove DCFH-DA that did not enter the cells. Flow cytometry was used for detection.

### Immunofluorescence analyses

NP cells were cultured in confocal dishes at a density of 100,000 cells per well in 24 wells. After incubation and culture for 24 h, the cells were treated according to the intervention protocol. After 24 h of intervention (PBS, TBHP, TBHP+DFOM, TBHP+CeO_2_, TBHP+DFOM@CeO_2_), the cells were fixed, permeabilized, and blocked. Subsequently, cells were incubated with specific primary antibodies (anti-GPX4(ET1706-45), anti-COL2A1(A19308), anti-MMP3(ab52915)) overnight at 4 °C. After 24 h, the cells were subsequently incubated with the corresponding secondary antibodies and phalloidin for 2 h at room temperature, and the nuclei were counterstained with DAPI for labeling. Representative images were acquired via laser confocal microscopy, and the fluorescence intensity was calculated via Image J software.

### Mitochondrial function assay

The mitochondrial membrane potential (ΔΨm) level was determined via a JC-1 staining kit. The NPCs were seeded in 24-well plates, and the culture was continued for 24 h after grouping intervention, and the culture medium was removed via aspiration. 1 mL of JC-1 staining working solution was added, and the mixture was thoroughly mixed. The cells were incubated in a cell incubator (37 °C, 20 min). During incubation, an appropriate amount of JC-1 staining buffer (1X) was prepared at a ratio of 4 mL of distilled water per 1 mL of JC-1 staining buffer (5 X) and placed in an ice bath. At the end of the incubation at 37 °C, the supernatant was removed by aspiration and washed twice with JC-1 staining buffer (1 X). 2 mL of cell culture medium was added. A fluorescence microscope was used for observation, and the fluorescence intensity was calculated via Image J software.

Cellular ROS were examined via MitoSOX Red (MedChemExpress, Monmouth Junction, USA). 100 μL of the working solution was added, and the mixture was gently shaken to completely cover the cells and incubated for 30 min. The dye working solution was blotted off, and the samples were washed with medium 3 times for 5 min each. A fluorescence microscope was used for observation and the fluorescence intensity was normalized to DAPI /Hoechst intensity, then calculated via Image J software.

### Micromass culture

The ability of nucleus pulposus cells to secrete ECM was detected by micromass culture. NP cells were seeded at a density of 6×10^7^ cells mL^-1^ in the center of a 6-well plate with 30 μL of cell suspension per well. After adhesion at 37 °C for 2 h, the cells were cultured with treatments in 5% high-glucose DMEM. The medium was changed every 2 days, and the cells were fixed on day 7. The extracellular collagen matrix was visualized via Alcian blue staining.

### Quantitative real-time polymerase chain reaction(qPCR)

After the cells were treated according to the intervention protocol, total RNA was extracted via an extraction kit, and the steps were performed according to the kit instructions (Vazyme, Nanjing, China). The cDNA was then prepared according to the instructions of the kit and the PCR thermocycler. Then reverse transcribed cDNA was diluted, and the RT-qPCR reaction mixture (10 μL of SYBR green, 0.5 μL of forward primer, 0.5 μL of reverse primer, 4 μL of ddH_2_O, and 5 μL of cDNA) was prepared according to the instruction. RT-qPCR was used to measure the mRNA expression of genes. GADPH was chosen as the reference gene, and the detailed primer sequences are provided in Table S3 (Supporting Information). The values were calculated via the 2^-ΔΔCt^ method.

### Western blotting analysis

After 24 h of intervention, PBS was added to wash the cells twice, and 300 μL of precooled RIPA lysis buffer was added to each well. We then quantified with protein using the BCA kit. Finally, the target protein concentration was calculated. After preparation of the gel, the sample was loaded according to the lane, the electrophoresis parameters were set to 80 V for 10 min and 150 V for 45 min, and the electrophoresis time was adjusted appropriately according to the actual situation until the bands were fully separated. A nitrocellulose membrane was used for the transfer, and the transfer condition was set at 350 mA for 50 min. After the membrane transfer was completed, an appropriate amount of blocking solution was added to the above NC membrane and blocked for 30 min at room temperature on a shaker. After blocking, the cells were incubated overnight with primary antibodies(primary antibodies, including anti-ADAMTS5 (ab41037), anti-Aggrecan (13880-1-AP), anti-ATP5A1 (ET1703-53), anti-β-Actin (66009-1-Ig), anti-COL2A1 (A19308), anti-GPX4 (ET1706-45), anti-MMP3 (ab52915), anti-MMP13 (18165-1-AP), anti-MTCO1 (HA500517), anti-NDUFB8 (ET7108-25), anti-SDHB (ET1706-30), anti-SOX9 (ab185966), and anti-UQCRC2 (HA721871) at 4 °C. The primary antibody was added, and the samples were washed three times with TBST for 10 min each time. Subsequently, the corresponding secondary antibodies were added and incubated for 1 h at room temperature on a shaker. The secondary antibodies were recovered and rinsed three times with TBST for 10 min each time. ECL developer solution was prepared and drip-added to the NC membrane. After incubation for 1 min, the gel imager was exposed and developed. Band gray values were quantified via Image J software.

### Rat IVDD model and treatment

The animal experiment was approved by the Ethics Committee of Soochow University (Approval No. SUDA20240326A01). Fifty male SD rats (400 g±20 g) were randomly divided into the sham group and the following four experimental groups: IVDD group, IVDD+DFOM group, IVDD+CeO_2_ group, and IVDD+DFOM@CeO_2_ group, with 10 rats in each group. The rats were anesthetized via an intraperitoneal injection of sodium pentobarbital, and the tails of the rats were disinfected with iodophor. Under the instructions of DR, the rats in the experimental group were punctured in the Co9-Co10 space with a 20 G needle, the depth of the needle was about 5 mm, and the needle was rotated 360°. After 30 s, the bleeding was stopped after the operation, and penicillin was injected intraperitoneally. In the IVDD group, IVDD+DFOM group, IVDD+CeO_2_ group, and IVDD+DFOM@CeO_2_ group, 2 μL of PBS, 2 μL of DFOM (20 μM), 2 μL of CeO_2_(20 μM), 2 μL of DFOM@CeO_2_(20 μM) were injected into the degenerative segment with a 33G needle once a week for 2 weeks from 0 week. At the end of the 4^th^ week and the 8^th^ week, 5 rats in each group were sacrificed for detection.

### Rat biosafety analysis

The rats were intraperitoneally injected with 1 mL of PBS or 1 mL of DFOM@CeO_2_, respectively, and divided into two groups according to the injection substance. Two weeks later, the samples (heart, liver, spleen, lung, and kidney) were taken for HE staining analysis.

### Histologic and Immunohistochemistry (IHC)

After fixation, the NPs and human NPs were decalcified. Subsequently, the tissues were dehydrated, infiltrated, and embedded in paraffin. Serial sectioning of 5 μm-thick sections was carried out, followed by staining with hematoxylin-eosin (H&E), safein O-fast green (SF), and immunochemical (IHC) stainings. IHC included COLII, MMP13, and GPX4. The morphology of the intervertebral discs was observed under a light microscope and the histological grade was determined.

### Ethics approval and consent to participate

The experimental procedures were performed under the approval of the Ethics Committee of First Affiliated Hospital of Soochow University, Suzhou, Jiangsu, China. Approval number is 2024550. The experiment procedures were recorded in National Health Security Information Platform—Medical Research Registration and Filing Information System (Record Number is MR-32-24-045370). They were in strict accordance with the Declaration of Helsinki (1964). Written informed consents were obtained from all participants.

### RNA Sequencing

NP samples from the animal models (IVDD group and IVDD+ DFOM@CeO_2_ group) were collected and cryopreserved according to the manufacturer's instructions, and subsequent RNA sequencing was performed by the company (Wekemo). Kyoto Encyclopedia of Genes and Genomes (KEGG) pathway enrichment analysis, Gene Ontology (GO) enrichment analysis, gene set enrichment analysis (GSEA), principal component analysis (PCA), and heatmap analysis were performed via the R and Bioincloud platform (https://www.bioincloud.tech).

### Statistical analysis

Independent experiments were conducted for all data (n = 3, 5, 6). Data are expressed as the mean ± standard deviation (SD). All statistical analyses were performed using SPSS version 25.0 (SPSS Corp., Armonk, NY, USA). For measurement data, unpaired two-tailed Student's t-tests were used to compare the two groups when the data exhibited a normal distribution. One-way ANOVA, followed by Tukey's posthoc test, was used to compare three or more groups. The Mann-Whitney and KruskalWallis tests were used for non-normally distributed data. Ranked data is tested using the Wilcoxon Signed-Rank Test. GraphPad Prism 8.0 (GraphPad Software Inc., San Diego, CA, USA) was used to design and generate the statistical charts. Statistical significance was set at p < 0.05.

## Supplementary Material

Supplementary figures and tables.
