# Supplementary material for: A strategy targeting ferroptosis for mitochondrial reprogramming and intervertebral disc degeneration therapy
Source: Theranostics. 2025 Aug 16;15(17):9159–78. doi: 10.7150/thno.117725 (PMC12439470; doi:10.7150/thno.117725)
Supplement: Supplementary file 1 — Supplementary figures and tables. [file thnov15p9159s1.pdf]

An Integrated Strategy Targeting Ferroptosis for Mitochondrial Reprogramming and Intervertebral Disc Degeneration Therapy

Tianyi Wu[1,2#], Yun Teng[1#], Dawei Song[1#], Yuqi Yang[2], Huaishuang Shen[1], Xiao Sun[1], Rui Chen[1], Leyu Zhao[1], Xianggu Zhong[1], Qi Yan[1], Junjie Niu[1*], Jun Ge[1,2*], Liang Cheng[2*], Jun Zou[1*]

[1] *Department of Orthopedic Surgery, The First Affiliated Hospital of Soochow University, Suzhou, Jiangsu 215000, China*

[2] *Institute of Functional Nano & Soft Materials (FUNSOM), Jiangsu Key Laboratory for Carbon-Based Functional Materials & Devices, Soochow University, Suzhou, Jiangsu, China*

***Corresponding authors. Email:** niujunjie1129@suda.edu.cn (J.N.); gejun115@suda.edu.cn (J.G.); lcheng2@suda.edu.cn (L.C.); jzou@suda.edu.cn (J.Z.)

[#]These authors contributed equally to this work.

Supporting Figures

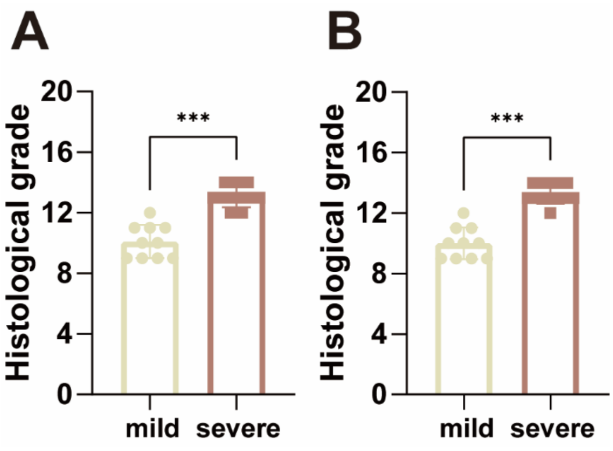

Figure S1. Histological grade of (A) Hematoxylin–Eosin (H&E) and (B) Safranin O-fast Green (S.O.) staining of different degenerated patient' groups.

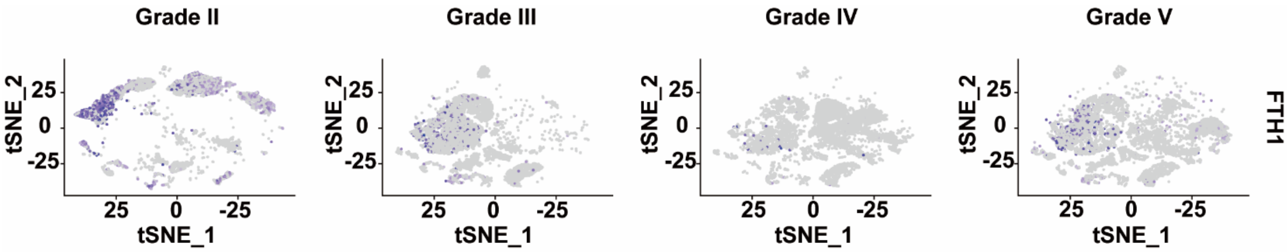

Figure S2. FTH1 immunohistochemical detection results in the nucleus pulposus tissues of different degeneration groups.

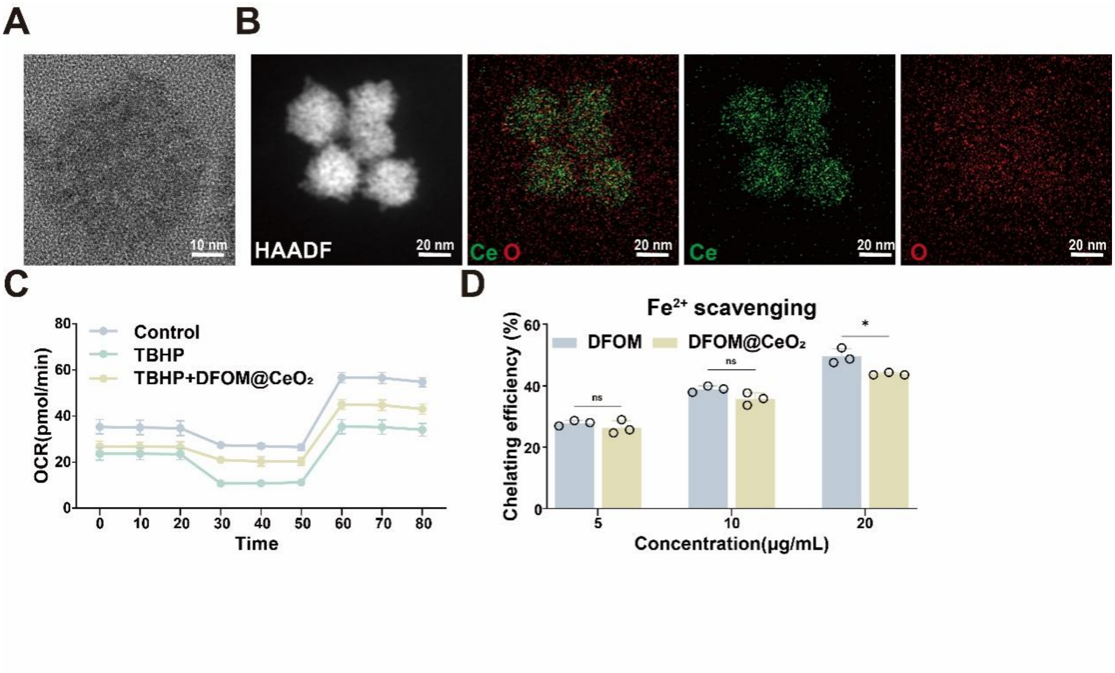

Figure S3. (A) Transmission electron microscopy images of DFOM@CeO_2. Scale bar: 10 nm. (B) The Energy Dispersive Spectroscopy (EDS) results of CeO_2. Scale bar: 20 nm. (C) The oxygen consumption rate (OCR) was measured using a XF96 SeaHorse Analyzer. (D) Comparison of iron ion clearance efficiency of DFOM and DFOM@CeO_2.

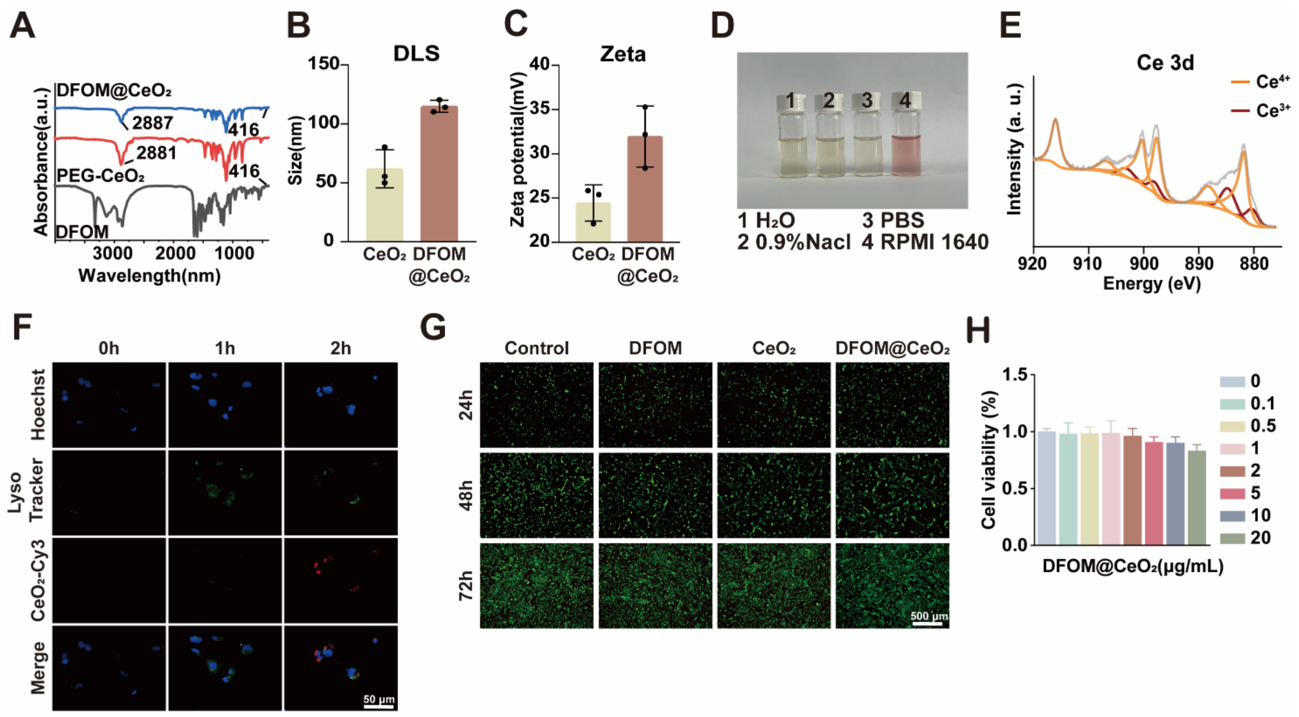

Figure S4. (A) Fourier transform infrared spectrogram of DFOM, PEG-CeO_2, and DFOM@CeO_2. (B) DLS of CeO_2 and DFOM@CeO_2. (C) Zeta potentials of CeO_2 and DFOM@CeO_2. (D) Photographs of the solubility of DFOM@CeO_2 in different solvents. (E) XPS of DFOM@CeO_2. (F) Intracellular transport of Cy3-labeled DFOM@CeO_2 in NPCs, Cy3-labelled DFOM@CeO_2 (red), lysosomes (green), and nuclei (blue). Scale bar: 50 μm. (G) Live/dead staining of NPCs co-cultured with DFOM@CeO_2 for 72 h. Scale bar: 500 μm (n = 3). (H) A CCK 8 assay was used to detect the cytotoxicity of DFOM@CeO_2 (n = 6).

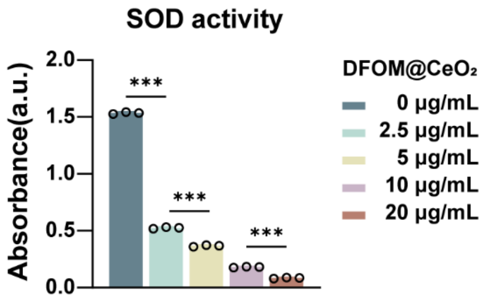

Figure S5. SOD activity of DFOM@CeO_2.

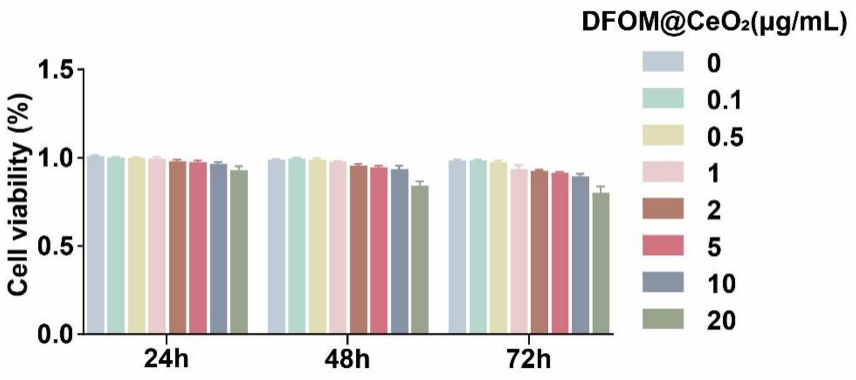

Figure S6.A CCK 8 assay was used to detect the cytotoxicity of DFOM@CeO_2 at 24h, 48h and 72h (n = 6).

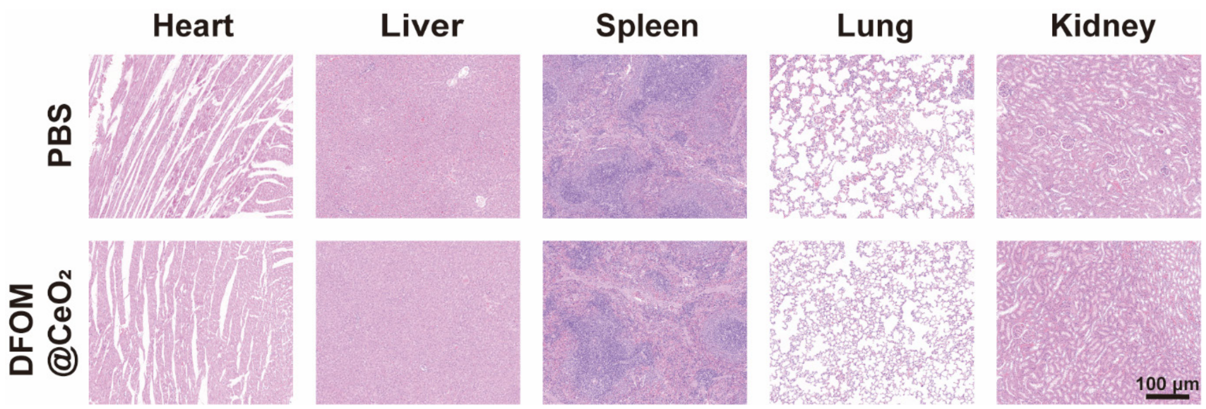

Figure S7. **In vivo biosafety analysis.** H&E staining of heart, liver, spleen, lung and kidney in DFOM@CeO_2-injected and normal rats. Scale bar:100 μm.

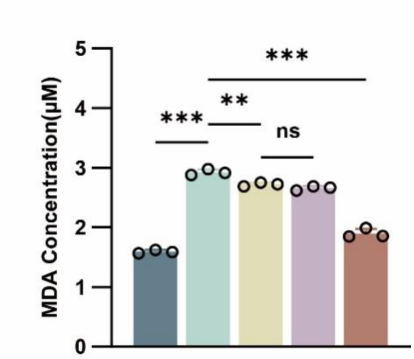

Figure S8. MDA results of NPCs treated with or without TBHP, TBHP+DFOM, TBHP+CeO_2, or TBHP+ DFOM@CeO_2 (n = 3).

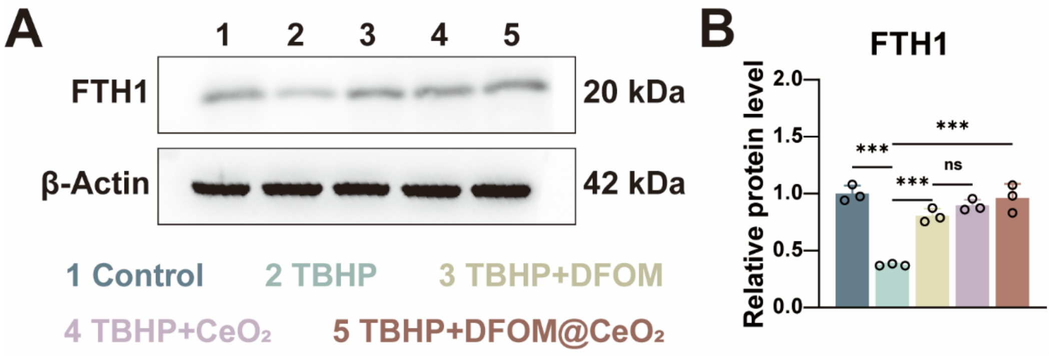

Figure S9. Western blot results and analysis of FTH1 in NPCs (n = 3).

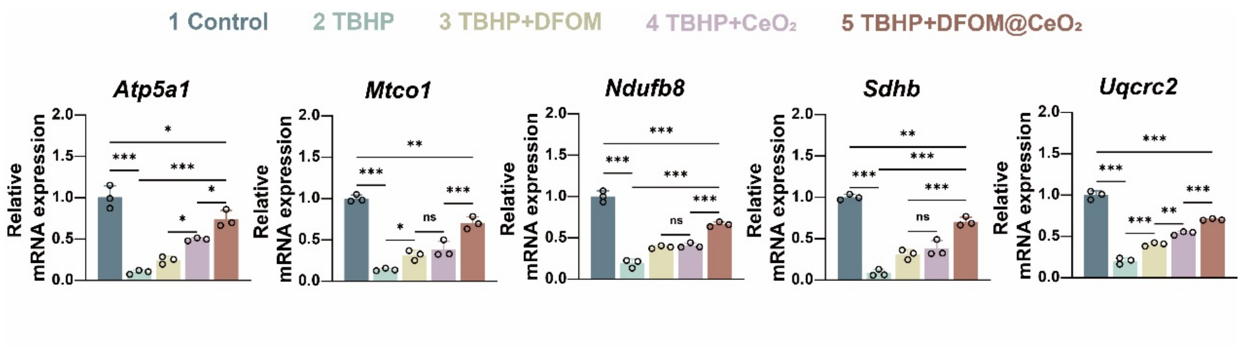

Figure S10. RT-qPCR results showing the mRNA expressions of *Atp5a1*, *Mtco1*, *Ndufb8*, *Sdhb* and *Uqcrc2* in NPCs treated with or without TBHP, TBHP+DFOM, TBHP+CeO_2, or TBHP+ DFOM@CeO_2 (n = 3).

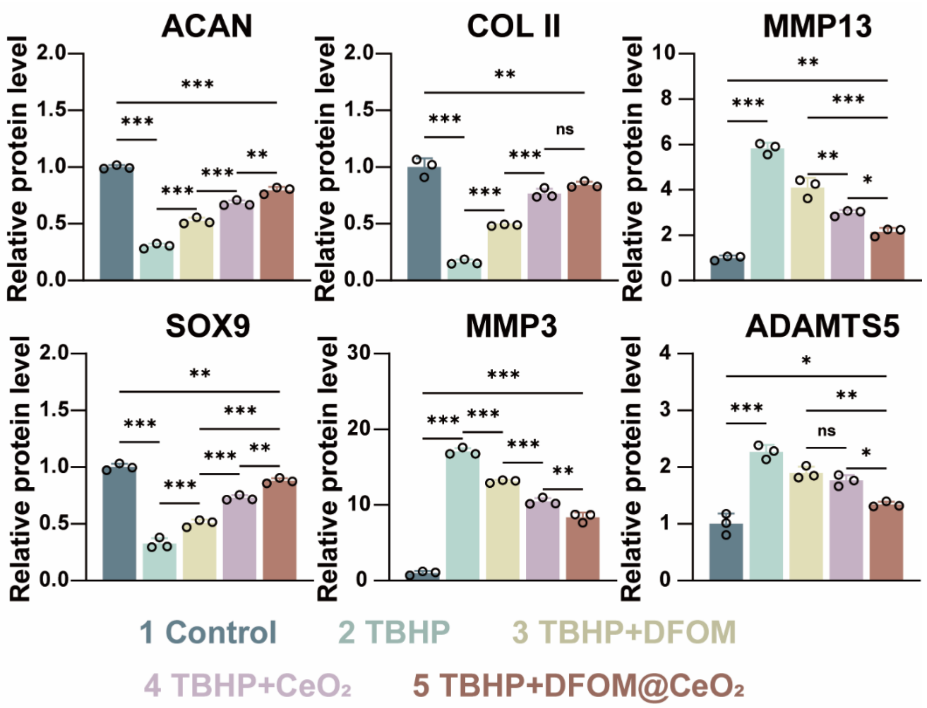

Figure S11. Western blot analysis of ACAN, COL II, MMP13, SOX9, ADAMTS5, and MMP3 in NPCs (n = 3).

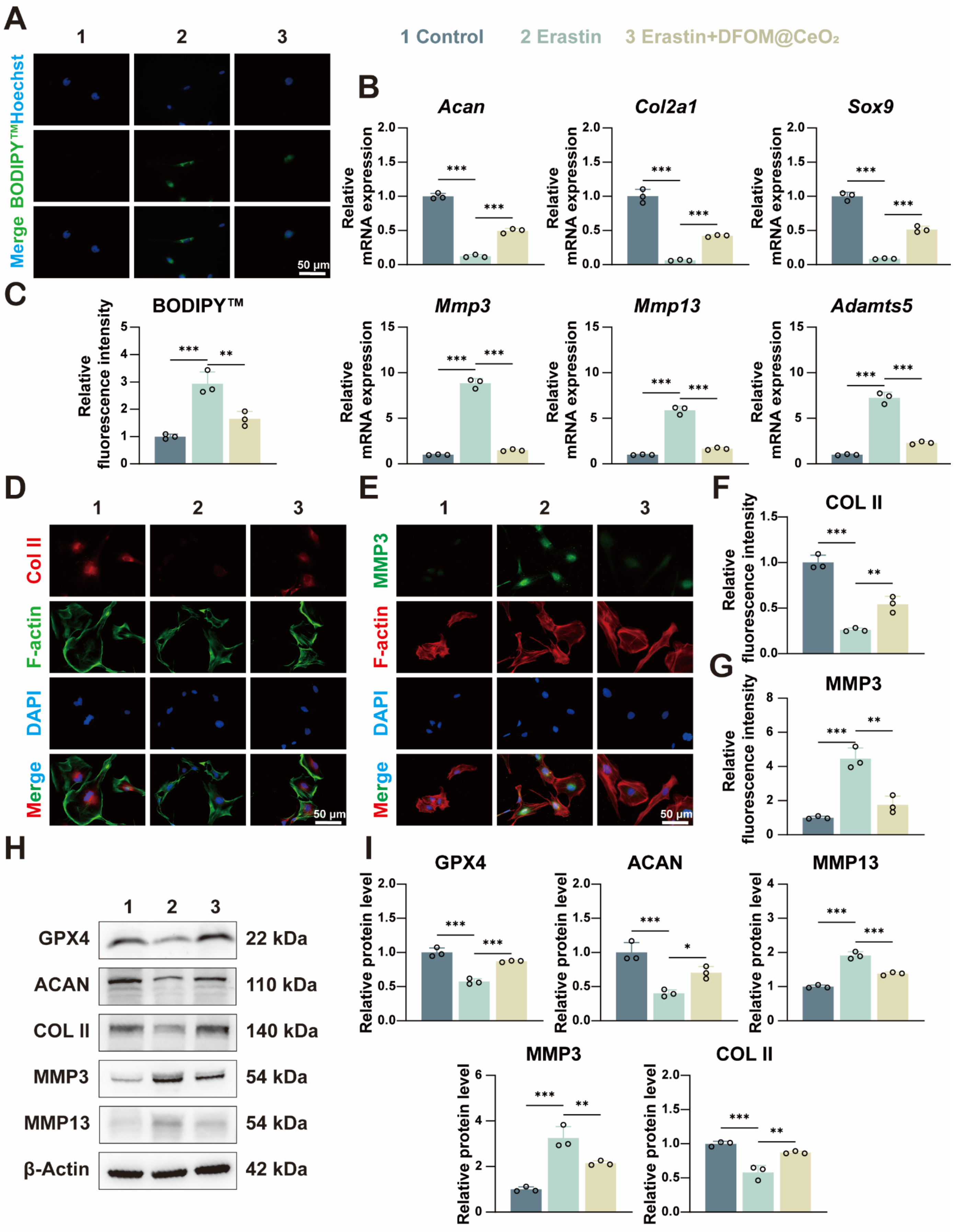

Figure S12. (A) Lipid peroxidation levels were detected by immunofluorescence staining for BODIPY^{TM} in NPCs. Scale bar: 50 μm (n = 3). (B) RT-qPCR results showing the mRNA expressions of *Acan*, *Col2a1*, *Mmp13*, *Sox9*, *Adamts5* and *Mmp3* in NPCs treated with or without Erastin, Erastin+DFOM@CeO_2 (n = 3). (C) Quantitative analysis of BODIPY^{TM} staining immunofluorescence (n = 3). (D) Immunofluorescence staining was used to analyze the expression of COLII in NPCs treated with or without Erastin,

Erastin+DFOM@CeO_2. Scale bar: 50 μm (n = 3). (E) Immunofluorescence staining was used to analyze the expression of MMP3 in NPCs treated with or without Erastin, Erastin+DFOM@CeO_2. Scale bar: 50 μm (n = 3). (F) Quantitative analysis of COLII staining immunofluorescence. (G) Quantitative analysis of MMP3 staining immunofluorescence. (H-I) Western blot analysis of GPX4, ACAN, COL II, MMP3, and MMP13 in NPCs (n = 3).

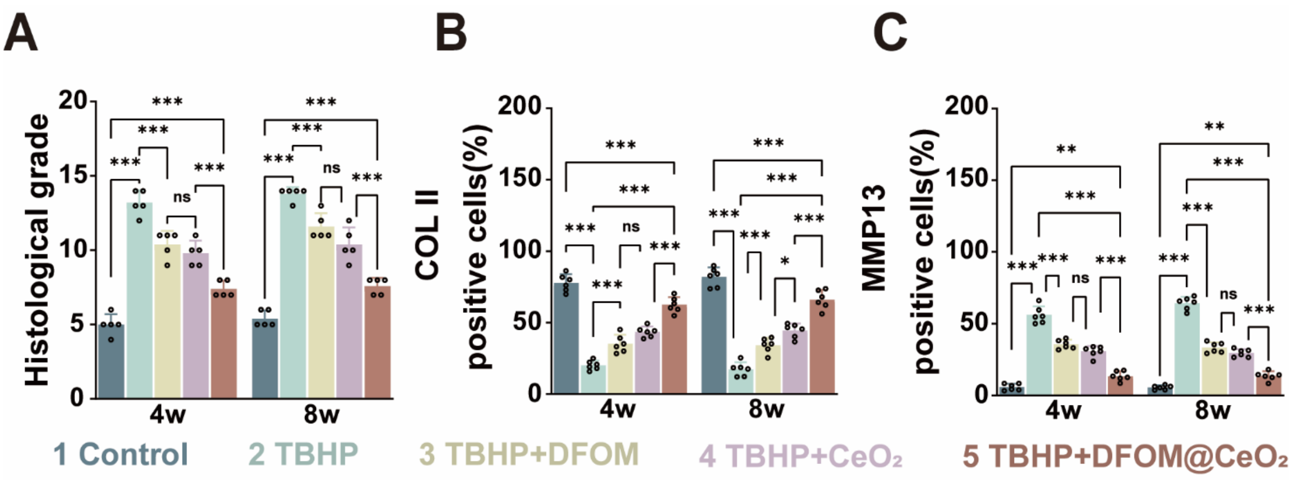

Figure S13. (A) Histological grade scores of Safranin O-fast Green (S.O.) staining of the IVDs at the 4th and 8th weeks. (B, C) Quantitative analysis of COLII-positive cells and MMP13-positive cells in the five groups at the 4th and 8th weeks (n = 5).

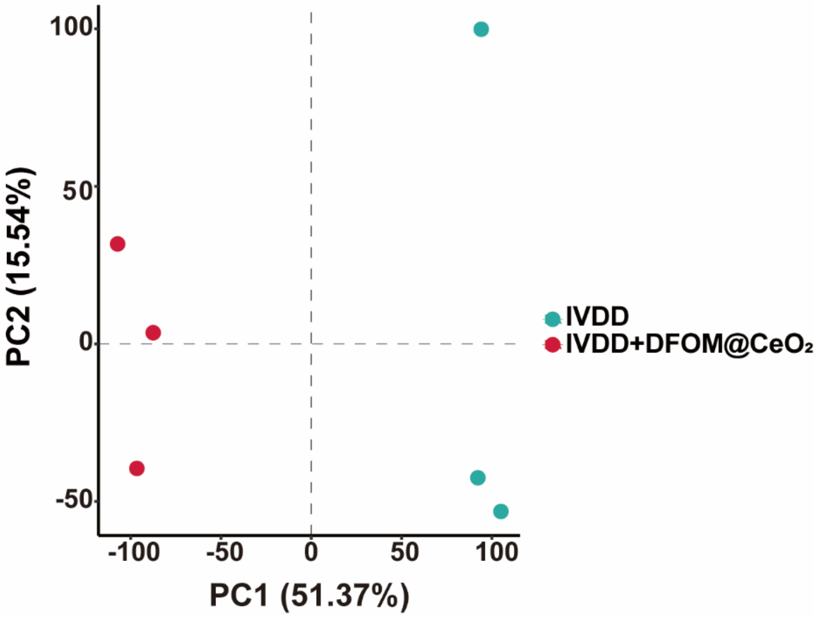

Figure S14. PCA analysis of samples.

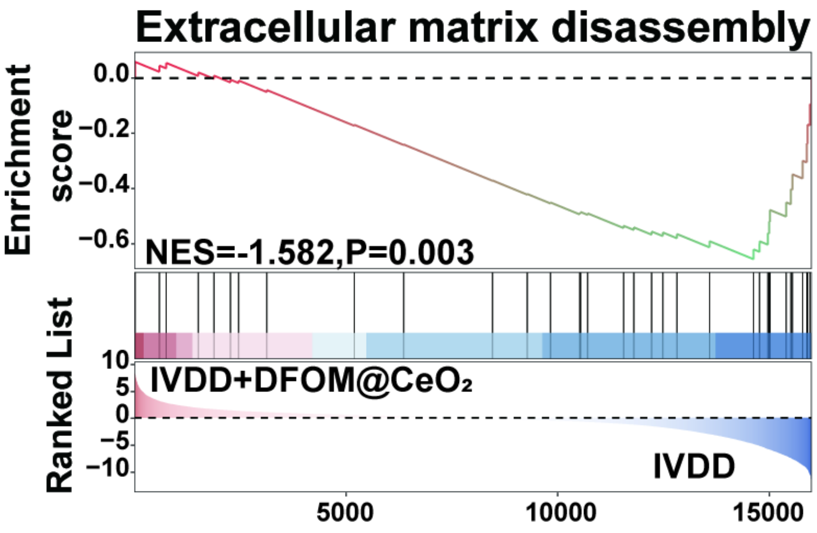

Figure S15. Extracellular matrix disassembly GSEA enrichment analysis based on GO datasets.

Table S1. **Clinical information of the patients.**

	Sex (Male/ Female)	Age (years)	Pfirrmann grade
Patient 1	Female	53	Grade II
Patient 2	Female	51	Grade IV
Patient 3	Female	47	Grade V
Patient 4	Female	61	Grade IV
Patient 5	Male	69	Grade V
Patient 6	Female	54	Grade III
Patient 7	Female	66	Grade II
Patient 8	Male	64	Grade V
Patient 9	Female	53	Grade II
Patient 10	Female	65	Grade IV
Patient 11	Female	59	Grade III
Patient 12	Female	63	Grade IV
Patient 13	Male	68	Grade II
Patient 14	Male	59	Grade III
Patient 15	Male	70	Grade V
Patient 16	Female	49	Grade III
Patient 17	Male	64	Grade IV
Patient 18	Male	67	Grade III
Patient 19	Male	43	Grade II
Patient 20	Female	71	Grade V

Abbreviations: PLIF, posterior lumbar interbody fusion

Table S2. **Demographic data of the 4 Groups.**

	Grade II	Grade III	Grade IV	Grade V	*P* value
Number of patients	5	5	5	5	
Age (years)	56.6±10.4	57.6±6.7	60.8±5.7	64.2±10.0	0.4957
Sex (Male/ Female)	2/3	2/3	1/4	3/2	0.4789
Operated segments					0.4411
L4-L5	4	3	2	1	
L5-S1	1	1	2	2	
L4-S1	0	1	1	2	
Number of operated segments					0.4789
1	5	4	4	3	
2	0	1	1	2	
Operation time (minutes)	182.4±63.2	200.4±42.1	186.0±79.0	218.4±34.9	0.7514
Intraoperative bleeding (milliliter)	211.0±115.9	167.0±100.0	214.0±76.1	192.0±120.7	0.8849

Table S3. Primer pairs for real-time PCR.

gene	Forward	Backward
Rat-ACAN	5′ －CAACCTCCTGGGTGTAAGGA-3′	5′-GTGTAGCAGATGGCGTCGTA-3′
Rat-Col2a1	5′ -TCCAGGTCTACAGGGAATGC-3′	5′ -CTTTTCCAGGAGCTCCCTCT-3′
Rat-MMP13	5′ -AAAGACTATCCCCGCCTCAT-3′	5′-TGGGCCCATTGAAAAAGTAG-3′
Rat-SOX9	5′ -CATCAAGACGGAGCAACTGA-3′	5′ -GTAGTGCGGAAGGTTGAGG-3′
Rat-MMP3	5′ -CCACAGAATCCCCTGATGTC-3′	5′-CTGACTGCATCGAAGGACAA-3′
Rat-Adamts5	5′ -GGGTTATACTGACGTTGTGAGG-3′	5′ -TCTAGTCTGGTCTTTGGCTTTG-3′
Rat-GAPDH	5′ -CCCCCAATGTATCCGTTGTG-3′	5′ -TAGCCCAGGATGCCCTTTAGT-3′
